# A Lachnospiraceae-dominated bacterial signature in the fecal microbiota of HIV-infected individuals from Colombia, South America

**DOI:** 10.1038/s41598-018-22629-7

**Published:** 2018-03-14

**Authors:** Homero San-Juan-Vergara, Eduardo Zurek, Nadim J. Ajami, Christian Mogollon, Mario Peña, Ivan Portnoy, Jorge I. Vélez, Christian Cadena-Cruz, Yirys Diaz-Olmos, Leidy Hurtado-Gómez, Silvana Sanchez-Sit, Danitza Hernández, Irina Urruchurtu, Pierina Di-Ruggiero, Ella Guardo-García, Nury Torres, Oscar Vidal-Orjuela, Diego Viasus, Joseph F. Petrosino, Guillermo Cervantes-Acosta

**Affiliations:** 1 División Ciencias de la Salud, Fundación Universidad del Norte, Barranquilla, Colombia; 2 División de Ingenierías, Fundación Universidad del Norte, Barranquilla, Colombia; 3 Alkek Center for Metagenomics and Microbiome Research, Department of Molecular Virology and Microbiology, Baylor College of Medicine, Houston, Texas USA; 4 IPS Medicina Integral, Barranquilla, Colombia; 5 Maestría en Estadística Aplicada, Universidad del Norte, Barranquilla, Colombia; 6 IPS de la Costa, Barranquilla, Colombia

## Abstract

HIV infection has a tremendous impact on the immune system’s proper functioning. The mucosa-associated lymphoid tissue (MALT) is significantly disarrayed during HIV infection. Compositional changes in the gut microbiota might contribute to the mucosal barrier disruption, and consequently to microbial translocation. We performed an observational, cross-sectional study aimed at evaluating changes in the fecal microbiota of HIV-infected individuals from Colombia. We analyzed the fecal microbiota of 37 individuals via 16S rRNA gene sequencing; 25 HIV-infected patients and 12 control (non-infected) individuals, which were similar in body mass index, age, gender balance and socioeconomic status. To the best of our knowledge, no such studies have been conducted in Latin American countries. Given its compositional nature, microbiota data were normalized and transformed using Aitchison’s Centered Log-Ratio. Overall, a change in the network structure in HIV-infected patients was revealed by using the SPIEC-EASI MB tool. Genera such as *Blautia*, *Dorea*, *Yersinia*, *Escherichia-Shigella* complex, *Staphylococcus*, and *Bacteroides* were highly relevant in HIV-infected individuals. Differential abundance analysis by both sparse Partial Least Square-Discriminant Analysis and Random Forest identified a greater abundance of Lachnospiraceae-OTU69, *Blautia*, *Dorea*, *Roseburia*, and Erysipelotrichaceae in HIV-infected individuals. We show here, for the first time, a predominantly Lachnospiraceae-based signature in HIV-infected individuals.

## Introduction

The health complications arising from HIV infection associated to developing acquired immunodeficiency syndrome impose a heavy physical and psychological toll on people^1^. Approximately 78 million people have been diagnosed with HIV, of whom ~35 million people have died. In 2015 alone, there were 36.7 million people living with HIV^1,2^. Among the 4 HIV groups, M is the most widely distributed group and is comprised of nine subtypes. Subtype C is responsible for about one-half of all global infections, while subtype B, the prevalent virus in Colombia, is the most widespread^3,4^.

A mucosal barrier integrated by the intestinal epithelium and the underlying immune system favors tolerance towards bacteria present in the intestine lumen^5–9^. Commensal bacteria of the intestinal microbiota also contribute to the mucosal barrier by competing for space and resources with potentially pathobiontic bacteria^10^. HIV exhibits a predilection towards the gastrointestinal tract immune system because of the presence of highly susceptible cells^11–13^. In HIV-infected individuals, substantial decrease in the levels of Th17 lymphocytes, and albeit to a less extent, in the levels of Foxp3(+) T lymphocytes are found within Peyer´s plaques and the intestine lamina^1,14–16^. Consequently, mucosal barrier is destroyed, and the permeability of the intestine is therefore increased. In turn, this results in a loss of tolerance and a chronic activation of CD4(+) T lymphocytes^1,13,17,18^.

Extrapolation from a population-level analysis on fecal microbiota composition suggested that 784 ± 40 genera of bacteria are capable of colonizing the human intestine^19^. Despite this enormous diversity, Falony *et al*. identified 17 bacterial genera that were common to 95% of individuals^19^. These genera, proposed to be the ¨core¨ of intestinal microbiota, include unclassified Lachnospiraceae, unclassified Ruminococcaceae, *Bacteroides*, *Blautia*, unclassified Erysipelotrichaceae, *Roseburia*, *Faecalibacterium*, unclassified Clostridiaceae, *Dorea*, *Alistipes*, unclassified Clostridiales, *Clostridium* XIVa, unclassified Veillonellaceae, unclassified Hyphomicrobiaceae, *Coprococcus*, *Clostridium* IV, and *Parabacteroides*^19^. Subsequently, this ¨core¨ was updated by removing *Alistipes*, *Clostridium* IV, and *Parabacteroides* after the incorporation of data obtained from 308 samples of Papua New Guinean, Peruvian, and Tanzanian origin^19^. Consequently, intestinal microbiota is influenced by the geographical location^20,21^. Environmental factors such as the type of diet or the presence of parasites, as well as to genetic factors, may contribute to such differences^20,22^.

Overall, the majority of studies found that HIV infection is associated with intestinal dysbiosis, characterized by increased numbers of Enterobacteriaceae and Enterococcaceae pathobionts, as well as by an enrichment of the genus *Prevotella*^23–27^. With the exception of three studies – two of which were conducted in China and the other based on a cohort in Uganda^27–29^ – all studies were conducted on western countries where the diets are rich in fats but low in fiber and complex carbohydrates^23–25^. Interestingly, the results obtained in the Uganda study substantially differ from the consensus observed in HIV-infected individuals from developed countries. Particularly, this study did not find any differences between HIV-infected and non-infected individuals with respect to the genus *Prevotella*^29^.

Here, in an observational and cross-sectional study, we assayed the fecal microbiota composition of a group of 25 individuals infected with HIV and a control group of 12 non-infected individuals similar in age, socioeconomical status, diet, and gender distribution. All individuals lived in the Caribbean coastal region of Colombia. To the best of our knowledge, this is the first study of this type that has been conducted in a South American country. We detect a fecal signature that differentiates HIV-infected individuals from non-infected individuals. This signature is characterized by an increase in bacteria belonging to the genera Lachnospiraceae-OTU69, *Blautia*, *Dorea* and Erysipelotrichaceae-OTU109 in the fecal microbiota of HIV-infected individuals. These results are distinct from previously reported signatures, likely due to the differences that our study population might have regarding diet and genetic makeup.

## Results

### Demographic characteristics of the HIV-infected and control groups and sequencing approach

We recruited 25 patients with chronic HIV infection living in Barranquilla and Cartagena – two closely located cities along the Colombian Caribbean Coast. These patients were being treated at two Healthcare Institutions which provide an integral care to HIV-infected patients (IPS Medicina Integral and IPS de la Costa). In addition, 12 non-infected individuals matched by gender distribution, body mass index (BMI), diet, and socioeconomic strata with the HIV-infected group were recruited from those who were blood donors of a blood bank (Table 1). Diet composition for both groups is shown as Supplementary Fig. S1.

**Table 1:** Descriptive data for subjects in the study.

	Non-Infected Control	HIV-Infected
Number of individuals	12	25
Age (years)	35.6 (18–54) Median: 39	29.4 (17–48) Median: 30
Male/Female ratio	7/5	15/10
Race	All Hispanics	All Hispanics
Body mass index (BMI kg/m^2^)	27.2 (23.5–33.9) Median: 26.0	24.1 (19.4–29.3) Median: 24.4
Socio-economic strata	Lower middle class: 33.3% Below poverty line: 66.7%	Lower middle class: 40% Below poverty line: 60%
Alcohol (low intake) (number of individuals)	2	1
Intermittent Smoking (number of individuals)	1	5
Viral load in blood plasma (copies/mL)	N/A	32,300.4 (0–240,000) Median: 58,698.8
CD4(+) T cell count (Cells/µL)	1,055.4 (501.2–2,619.3) Median: 841	546.5 (39.3–1,012.4) Median: 528.9
CD4/CD8 ratio	1.802 (0.76–2.60) Median: 1.80	0.606 (0.03–1.80) Median: 0.51
Percentage of Th-17 lymphocytes (%)	1.9 (0.4–4.4) Median: 1.55	2.5 (1.1–5.3) Median: 2.0
Percentage of activated CD4(+) T cells (%)	2.62 (1.0–3.9) Median: 2.6	9.6 (1.0–27.1) Median: 8.0
HIV-Low % activated CD4(+) T cells	NA	N = 4 (1− < 5%)
HIV-Medium % activated CD4(+) T cells	NA	N = 11 (5–10%)
HIV-High % activated CD4(+) T cells	NA	N = 10 (>10%)
Percentage of activated CD8(+) T cells (%)	8.8 (2.2–32.4) Median: 6.65	37.6 (4.0–82.4) Median: 35.3

Data is presented as “mean(range) and median” for age, BMI, viral load, CD4(+) T cell count, CD4/CD8 ratio, percentage of Th17 lymphocytes, percentage of activated CD4(+) T cells, and percentage of activated CD8(+) T cells.

La Rosa *et al*.^30^ provided calculations for the power achieved in taxonomic-based human microbiome studies. La Rosa *et al*. found that studies with 5% significance level, 20,000 reads per subject and 25 subjects will have a power over 97%. In our study, we performed 30,000 reads per subject and included 37 individuals (25 HIV-infected and 12 controls). Kelly *et al*.^31^ indicated that sample sizes above 10 individuals per group (ntotal = 20) likely afforded adequate statistical power (i.e., of at least 90%) for the primary outcome measure in microbiome studies. Thus, our sample size was enough to detect the differences to be reported.

We excluded those individuals who had a BMI ≤ 18.5 kg/m^2^, showed signs of active liver disease (determined by alanine aminotransferase levels), or took antibiotics during the last 30 days previous to sample collection. Three of the HIV-infected patients had consumed antibiotics 31 to 60 days prior to sample collection. More specifically, patient V.02 had undergone one cycle of trimethoprim/sulfamethoxazole, patient V.03 one cycle of dicloxacillin, and patient V.07 one cycle of ampicillin/azithromycin.

All recruited patients were recently diagnosed and they were in stage V according to Fiebig classification criteria. When recruited patients were clinically stable and had no acute illness. They were still in the assessment period for HAART assignment.

According to the Bristol index of stool consistency, the samples of 22 out of the 25 samples from HIV-infected patients were classified as normal (stool type 3 or 4), while samples V.15 and V.16 were classified as type 2 - slightly constipated, and only sample V.19 as type 6 - semi-liquid. All fecal samples obtained from the control group were classified as normal.

About 13.4 million pairs of reads were generated after sequencing the V4 region of 16S rRNA gene using the Illumina-MiSeq. 2 × 250 bp. After proper quality control and demultiplexing, the number of reads was reduced to 1,420,474, being equivalent to 264,027 unique sequences. Singletons and chimeras were also removed. This yielded 1,286,018 reads, or 726 operational taxonomic units (OTUs) after applying a similarity cut-off of 97% to define species. Approximately 29,856 reads were analyzed per sample (Supplementary Fig. S2).

There were no differences between HIV-infected and control groups in terms of observed species as well as in Chao and Shannon indexes (Supplementary Table S1). The complete list of the OTUs at the genus level is shown in Supplementary Table S2. The bacterial counts per each OTU at the genus level are shown in Supplementary Tables S3–S6.

### The intestinal microbiota of HIV patients follows a universal pattern

**Figure 1:**
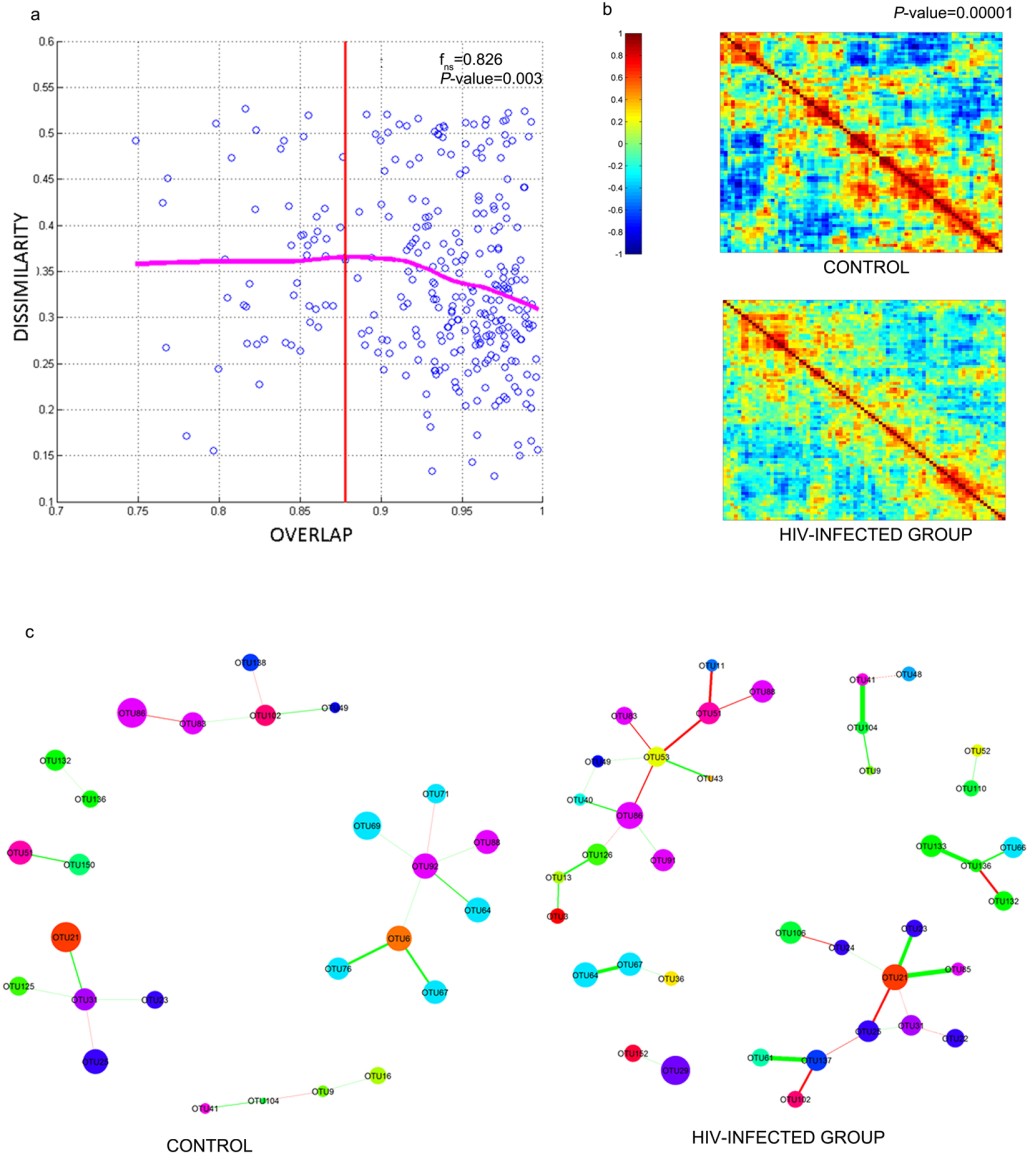
Network structure of the microbial communities found in HIV-infected and control individuals. (**a**) The fecal microbiota of HIV-infected individuals follows a pattern of universality as described by Bashan *et al*.^32^. Each point represents the comparisons between the samples of two individuals. The fraction of shared bacteria (*overlap*) is plotted against the dissimilarity index, which was evaluated based on the square root of the Jensen–Shannon divergence. The dissimilarity index calculates the distance between pairs of individuals in terms of abundance of shared species. The dissimilarity-overlap curve (DOC) is represented by a pink curve and was calculated using the robust LOWESS method. The fraction of points beyond the inflection point (O_C_) of the DOC curve – the point at which the curve shows a negative slope - was calculated by: ${{f}}_{{ns}}=\frac{{number}\,{of}\,{pair}\,{samples}\,{with}\,O\, > {{O}}_{{c}}}{{total}\,{number}\,{of}\,{sample}\,{pairs}}$. The *P*-values are finally calculated as the fraction of bootstrap runs resulting with non-negative slopes. (**b**) Heat map of Pearson Correlation of HIV-infected individuals and non-infected control group. We performed a Jennrich test to calculate the p-value associated to the correlation structures between HIV-infected patients control group. (**c**) Differences in the microbial networks of fecal samples obtained from HIV-infected and control individuals. Using the SPIEC-EASI MB method, networks were constructed from the table of clr-transformed OTUs.

Bashan *et al*.^32^ reported that as more genera are shared between people, each shared genus tend to have the same abundance in those individuals, reflecting a universal dynamics emerging among the various genera and species. This dynamic is lost in the presence of a strong destabilizing factor. This finding prompted us to investigate whether immune dysregulation due to HIV infection disrupted the dynamics of intestinal microbiota. Such immune dysregulation was evidenced by the inverse CD4/CD8 ratio and an increase in the percentage of activated CD4 T lymphocytes (Table 1). We built a Dissimilation-Overlap Curve (DOC) for each pair of samples being compared, and calculated the fraction of genera that is shared versus how dissimilar the abundance is in those shared genera (“dissimilarity index”). DOC showed that 82.6% of sample pairs were located after the inflection point – the point at which the curve started taking a negative slope (Fig. 1a), meaning that the intestine of these individuals must be colonized by microorganisms that build relationships with each other, and thereby favor network building. The P-value of 0.003 was calculated as the fraction of bootstrap runs (n_boot = 1000) resulting with non-negative slopes.

### Changes in the correlation patterns and network architecture of intestinal microbiota of HIV-infected patients

Since DOC reflects an underlying network^32^, we compared the structure of correlation- and precision-matrices of the microbiota from HIV-infected individuals to the microbiota from the control group. Since the data had a compositional architecture, we first transformed it using Aitchison´s centered log-ratio (clr) procedure^33–37^. We then calculated the Pearson’s correlation matrices using heat maps (Fig. 1b). A Jennrich test^38^ showed that the correlation structure for HIV-infected patients was significantly different from that of the control group (P-value < 0.00001). The microbiota of individuals from the control group presented more correlations compared to those of the HIV-infected group.

As correlation matrices included both direct and indirect relationships. We used the SPIEC-EASI-MB method^39^ to construct a network assessing only direct relationships. A sparse network was built to highlight the more stable relationships (Fig. 1c). HIV-infected group displayed a far more enriched network architecture in terms of direct relations between OTUs. We also built networks whose nodes were fixed in their position to clearly show the differences between the network structures of the HIV-infected and control groups (Supplementary Fig. S3a). In HIV-infected group, seven clusters were found, two of which were composed by 14 and 11 OTUs, respectively. Meanwhile, six clusters were identified in the control group; the maximum number of OTUs observed for one cluster was eight (Supplementary Fig. S3b). In addition, both frequency-vs-edges and frequency-vs-degree plots confirm the higher correlated structure in terms of direct relations between nodes in the HIV network (Supplementary Fig. S3c,d).

When comparing both networks, we found differences in which genera took the role of major nodes and their relationships. For instance, the *Bifidobacterium* (OTU6), *Subdoligranulum* (OTU92) and *Enterobacter* (OTU132) genera that act as nodes in the control group disappeared in the HIV-infected group. In the control group, the genus *Bifidobacterium* establishes a mutualistic relationship with the genus *Dorea* (OTU67), while the genus *Subdoligranulum* establishes a weak relationship with the genus *Blautia* (OTU64). However, in HIV-infected patients, as the nodes corresponding to the *Bifidobacterium* and *Subdoligranulum* genera disappeared in HIV-infected group, a strong mutualistic relationship formed between two genera of the Lachnospiraceae family - *Blautia* and *Dorea*.

As the genus *Enterobacter* lost its major node property in HIV-infected patients, *Yersinia* (OTU136) established strong mutualistic relationships with the *Coprococcus* (OTU66) and the *Escherichia-Shigella* complex (OTU133). Both *Yersinia* and *Escherichia-Shigella* belong to the family Enterobacteriaceae. Although the genera *Staphylococcus* (OTU41) and *Allobaculum* (OTU104) are preserved in both groups, these genera became more collaborative in the HIV-infected group. Furthermore, the genus *Bacteroides* (OTU21) established associations with *Butyricimonas* (OTU23) from the Bacteroidaceae family, and *Flavonifractor* (OTU 85) from the Ruminococcaceae family in HIV-infected group.

In summary, the microbiota network structure changed in patients infected with HIV, making the genera *Blautia* (OTU64), *Dorea* (OTU67), *Yersinia* (OTU136), *Escherichia-Shigella* (OTU133), *Staphylococcus* (OTU41), and *Bacteroides* (OTU21) relevant in the HIV network.

### HIV-infected individuals have a distinct bacterial signature compared to control individuals

**Figure 2:**
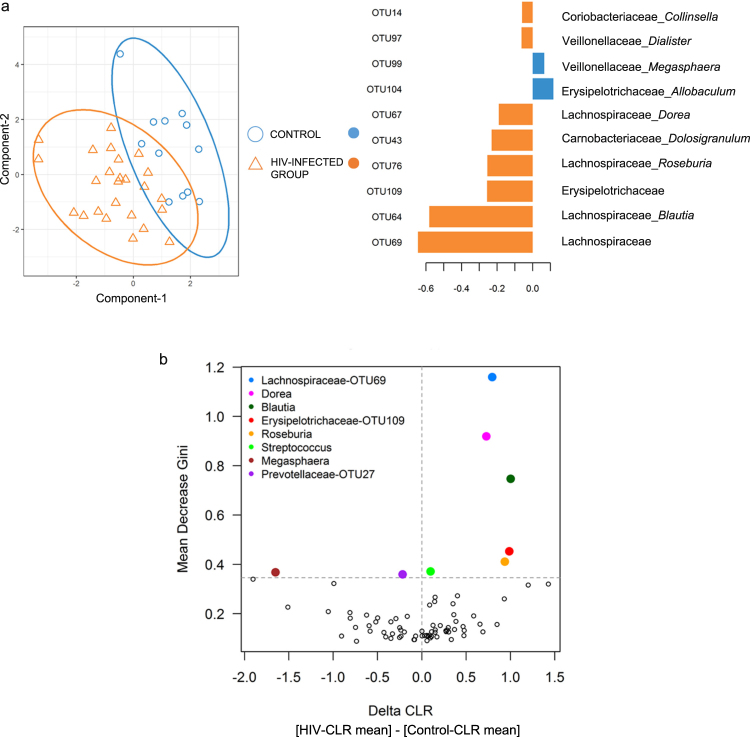
Identification of an OTU signature associated with HIV-infected individuals. (**a**) PCA analysis of the sPLS-DA model fed with clr-transformed OTUs (left), and the resulting contribution of OTUs of component 1 (right). (**b**) OTU importance according to the random forest method. The Mean Gini Decrease of each OTU is plotted versus the delta of the CLR averages for each OTU (i.e., for each OTU, the ΔCLR was calculated subtracting the CLR mean of the HIV-infected group from the CLR mean of the control group).

Using the sparse-partial least squares-discriminant analysis (sPLS-DA) technique^40,41^, we found that the HIV-infected group separate from the control group (Fig. 2a). In order of decreasing influence, the former group consists of an undefined genus from the Lachnospiraceae family (OTU69), *Blautia* (OTU64), an undefined genus from the Erysipelotrichaceae family (OTU109), *Roseburia* (OTU76), *Dolosigranulum* (OTU43), *Dorea* (OTU67), *Dialister* (OTU97), and *Collinsella* (OTU14) (Fig. 2a). These genera belong to the families Lachnospiraceae (OTU69, *Blautia*, *Roseburia*, and *Dorea*), Erysipelotrichaceae (OTU109), Carnobacteriaceae (*Dolosigranulum*), Veillonellaceae (*Dialister*), and Coriobacteriaceae (*Collinsella*). Consequently, the order Clostridiales clustered most of the genera identified in the signature.

We used random forest (RF) classifiers^42^- a machine-learning technique – to evaluate whether the signature found by sPLS-DA could be replicated (Fig. 2b). The Mean Decrease Gini (MDG) criterion was used as a measure of relative importance for each OTU. We found that the results generated through RF nicely correspond with those of sPLS-DA. Five of the eight genera identified by sPLS-DA occupied the top positions having the highest MDG. In decreasing order these were Lachnospiraceae-OTU69 (MDG = 1.159), *Dorea* (MDG = 0.918), *Blautia* (MDG = 0.747), Erysipelotrichaceae-OTU109 (MDG = 0.453), and *Roseburia* (MDG = 0.411). The MDG values of *Dialister*, *Dolosigranulum* and *Collinsella* fell below the 90^th^ percentile, with having MDG values of 0.32, 0.316 and 0.26 respectively. The Δ-clr showed that the five concordant genera between the sPLS-DA and RF techniques were more abundant in the HIV-infected group. Interestingly, three of these genera (Lachnospiraceae-OTU69, *Blautia* and *Dorea*) belong to the Lachnospiraceae family.

### Specific bacteria are associated with the severity of HIV infection

**Figure 3:**
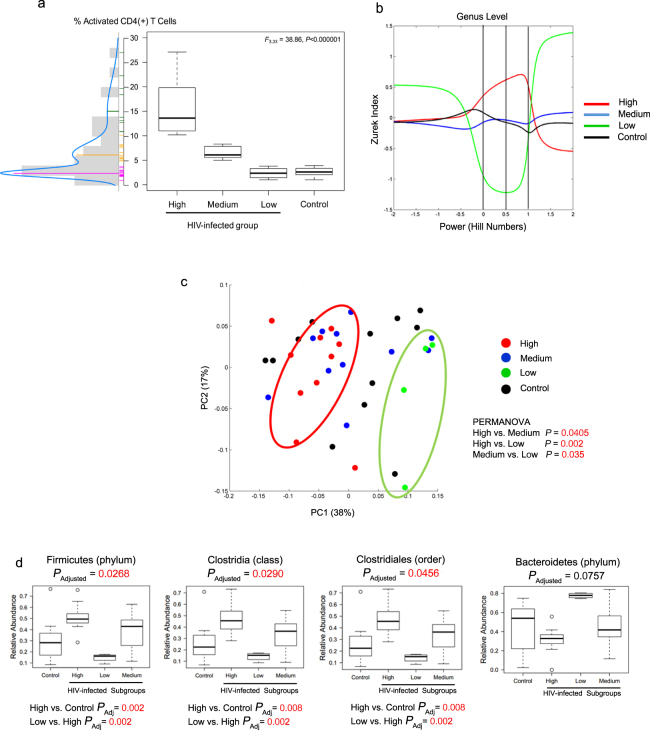
Identification of OTU signatures associated with CD4(+) T cell activation status. (**a**) Subgroups of HIV-infected individuals according to their percentage of CD4(+) T cell activation. ANOVA-based analysis was performed to calculate statistical difference among groups. (**b**) Diversity indices, based on the intrinsic relationship of each one with respect to the transformation of Hill numbers, are plotted continuously on the x-axis. The Chao, Shannon and Simpson indices are shown when the value of the Hill numbers are -2, 0.5 and 2, respectively. The homogeneity with respect to the distribution of OTU abundance is an inverse function of the Simpson index. The richness of the OTUs is a direct function of the Shannon index. (**c**) PCA plot of β-diversity using the weighted UNIFRAC metric. PERMANOVA was used to estimate P-value. (**d**) Relative abundance of Firmicutes and Bacteroidetes in HIV-infected subgroups. Statistical difference among groups was estimated using ANOVA and *P*_Adjusted_ was calculated using false discovery rate and Bonferroni. Statistical difference between subgroups was estimated using Tukey least significance difference test with 95% confidence intervals (CIs) and P-value was adjusted by multiple comparisons.

From the assessed immune parameters, both the percentage of activated CD4(+) T cells and CD4/CD8 ratio were the only factors able to discriminate between the HIV-infected and the control groups. Since CD4 loss is in great extent driven by immune activation^43^, we assessed CD4(+) T cell activation based on the coexpression of HLA-DR and CD38. We represented the percent activation values as a continuous function and identified three distinct subgroups in the HIV-infected group using a mixture of Gaussian distributions (Fig. 3a). Subgroup “High” included 10 HIV-infected individuals with percentage of activated CD4(+) T cells levels greater than or equal to 10%; subgroup “Medium” consisted of 11 patients whose levels were ≥5% but <10%, and subgroup “Low” was comprised of four patients whose percentage values were comparable to those of the control group (i.e., <5%). The bacterial counts per each OTU at the genus level for the HIV subgroups and the control group are shown in Supplementary Tables 3–6 ANOVA-based analyses showed that the average percentage differed among subgroups (*F*_3,33_ = 38.86, *P* < 0.00001). This result was subsequently confirmed using a Tukey least significance difference test with 95% confidence intervals (CIs) was performed (Supplementary Table S7).

We found that HIV subgroups differed in indices of α-diversity (Fig. 3b). In particular, the Chao, Shannon, and Simpson indices corresponded to the *x*-axis values of −2, 0.5 and 2, respectively. Based on Shannon and Simpson indexes, the subgroup-High showed a greater richness, homogeneity, and evenness in terms of bacterial OTUs compared with subgroup-Low (p = 0.0039) (Supplementary Fig. S4). The highest Simpson indices and the lowest Shannon index score in subgroup-Low implied that a small share of the OTUs carried most of the bacterial counts (p = 0.0039) (Supplementary Fig. S4). Nonetheless, this result should be taken with caution as this subgroup was comprised of only a small number of individuals.

We calculated β-diversity metrics by employing UNIFRAC weighted criteria and the principal coordinate analysis (PCoA) ordination method (Fig. 3c). The control group was distributed throughout the entire plot space, while the subgroups-High and -Low were located at the opposite sides on first principal coordinate (PERMANOVA p-value = 0.002). Although subgroup-Medium made a gradient between the two other subgroups, it was still significantly separated from subgroup-High (PERMANOVA p-value = 0.045) and from subgroup-Low (PERMANOVA p-value = 0.035).

We then probed the Firmicutes/Bacteroidetes (F/B) ratio across HIV-infected subgroups. We found an F/B higher than 1 in 90.0%, 54.4%, and none of the individuals from subgroups High, Medium, and Low, respectively. These suggest that the abundance of Firmicutes (*P*_ANOVA-FDR_ = 0.0268), Clostridia (*P*_ANOVA-FDR_ = 0.0290) and Clostridiales (*P*_ANOVA-FDR_ = 0.0456) were significantly different among subgroups. Using Tukey’s test adjusted for False discovery rate (FDR), we found that subgroup-High had a significantly greater proportion of Firmicutes, Clostridia and Clostridiales than both the control group (*P*_Bonferroni_ = 0.008) and the subgroup-Low (*P*_Bonferroni_ = 0.002) (Fig. 3d). In contrast, for Bacteroidetes, we discriminated among subgroups only at the phylum level (*P*_ANOVA-FDR_ = 0.0378). Particularly, subgroup-Low had significantly larger proportion of Bacteroidetes compared to the control (*P*_Bonferroni_ = 0.0428) and subgroup-High (*P*_Bonferroni_ = 0.0025) (Fig. 3d).

**Figure 4:**
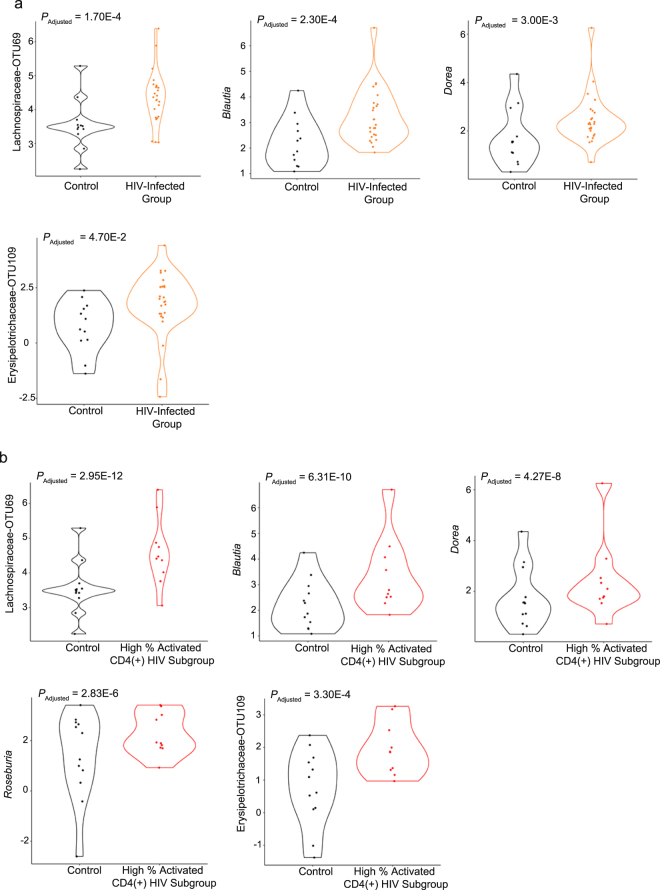
MetagenomeSeq analysis. OTUs that were both identified as part of the signature by sPLS-DA/random forest and had a statistically significant P_adj_ value (Padj < 0.05) are shown. For better visualization, CLR values were used for plotting the abundance of each OTU. The P_adj_ values are shown in each respective panel. (**a**) Comparison of the control group with the HIV-infected group. (**b**) Comparison of the control group with the HIV-infected subgroup with high percentage of activated CD4(+) T cells.

In our report, no difference was found in terms of Th17 and Treg between HIV-infected patients and control individuals. A clustergram in Supplementary Fig. S5 shows that both Th17 and Treg have no correlation with OTUs that separates control group and HIV-infected group. We employed the MetagenomeSeq tool^44^ to assess whether the bacteria in the previously identified signature could discriminate between subgroups. This tool operates acceptably for datasets having a sparse and compositional nature^45–47^ (Fig. 4). When comparing the HIV-infected group as a whole with the control group, four of the five genera from the signature were statistically significant: Lachnospiraceae–OTU-69 (*P*_adjusted_ = 0.00017), *Blautia* (*P*_adjusted_ = 0.00023), *Dorea* (*P*_adjusted_ = 0.003) and Erysipelotrichaceae-OTU109 (*P*_adjusted_ = 0.047) (Fig. 4a). Supplementary Table S8 shows the complete list of those OTUs with *P*_adjusted_ < 0.05. Comparison between subgroup-High and the control group revealed 32 genera with *P*_adjusted_ < 0.010. All genera belonging to this signature were statistically significant: Lachnospiraceae-OTU69 (*P*_adjusted_ = 2.95E-12), *Blautia* (*P*_adjusted_ = 6.31E-10), *Dorea* (*P*_adjusted_ = 4.27E-8), *Roseburia* (*P*_adjusted_ = 2.83E-6) and Erysipelotrichaceae-OTU109 (*P*_adjusted_ = 0.00033) (Fig. 4b). Supplementary Table S9 shows the top-half of those OTUs with *P*_adjusted_ < 0.05.

## Discussion

Here, we conducted an observational, cross-sectional study aimed to determine how the fecal bacteria of HIV-infected individuals differs from that of non-infected individuals in a Colombian setting. To the best of our knowledge, this is the first time that a study of this type has been conducted in a South American country. Our results indicate that the microbial fecal community of HIV-infected patients established associations among themselves which allowed us to probe for network building. Such relationships can respond in a dynamic way to environmental factors such as variations in diet and/or host health^32,48,49^.

Since sequencing data is of a compositional nature, we applied Aitchison’s centered log ratio-transformation (clr) to avoid spurious associations arising from such compositional structure and consequently be able to find true associations and correlations^33^. We found that the network structure of HIV-infected individuals highlights a dynamic in which the *Blautia*, *Dorea*, *Yersinia*, *Escherichia-Shigella*, *Staphylococcus* and *Bacteroides* genera all appear as nodes.

By applying the sPLS-DA and random forest statistical methods on a matrix of clr-transformed microbiota data^33^, we found a genus-level signature of OTUs discriminating between HIV- and control individuals. This signature consists of the genera from both the Lachnospiraceae family (Lachnospiraceae-OTU69, *Blautia*, *Dorea, Roseburia*) and the Erysipelotrichaceae family (Erysipelotrichaceae-OTU109).

In terms of α-diversity, the individuals who had the lowest percentage of activated T cells had a heterogeneous distribution dominated by very few bacterial species. In contrast, the HIV-infected individuals belonging to subgroup-High had a homogeneous distribution comprised of a more diverse bacterial population. Α-diversity indexes reported by other studies have covered the entire spectrum of possibilities^23,26–28,50,51^, which might be due to the heterogeneity of the course of infection or to the immunological status of the individuals.

Overall, the bacterial signature we observed in HIV-infected patients can be summarized as an increase in the relative abundance of the genera Lachnospiraceae-OTU69, *Blautia*, *Dorea*, and Erysipelotrichaceae-OTU109. Genera within the Erysipelotrichaceae family have consistently been reported as part of the bacterial signature associated with the microbiota dysbiosis in HIV-infected individuals^23–25,27,52^. Erysipelotrichaceae positively correlated with TNF-α and chronic intestinal inflammation in simian immunodeficiency virus-infected animals, suggesting its considerable potential to cause host perturbations^53^. Although most of the studies found Lachnospiraceae to be associated to non-infected individuals^24,28,50,54,55^, that was not our case. Although *Blautia*-*Dorea* pair has a beneficial effect for the intestinal mucosa^56,57^, their presence has been positively correlated with intestinal permeability in individuals with alcohol dependence or who suffered multiple sclerosis relapse episodes^58–60^, and is one of the colitogenic genera favoring Crohn´s disease development^61^.

In contrast with other studies^23,25–28,50,52^, we did not find an increased abundance in the *Prevotella* genus or from genera belonging to the Enterobacteriaceae or Enterococcaceae families. However, enteropathogenic bacteria may appear as the course of infection progresses as is shown by *Yersinia* and the *Escherichia*-*Shigella* appearing in HIV-infected group network structure. Overall, such dissimilarities might be due to differences in gut microbiota steady-state prior to infection-induced destabilization, disease status, the environmental factors, and environmental interaction with the unique genetic makeup of this hispanic population.

HIV-infected individuals showed a reduced capacity to produce IgA and IgG against new antigens derived from incoming Firmicutes and Proteobacteria^58^. This observation seemed to be compatible with our findings. Moreover, the levels of Firmicutes seemed to increase as individuals showed higher percentages of activated CD4(+) T cells. As such, it would be interesting to investigate whether such expansion of Firmicutes communities could be explained by a lack of IgA and IgG updating in the face of a dynamic microbiota^58^.

Some genera of Lachnospiraceae family may produce SCFAs^62^. Under healthy conditions, SCFAs range from 70 to 140 mM in the proximal colon and from 20 to 70 mM in the distal colon^57^. No study has actually determined whether HIV-infected individuals microbiota has a diminished capacity to produce SCFAs. In our case, *Blautia* is capable of producing acetate from the end products of the glucose metabolism of *Dorea*^63^. Moreover, Erysipelotrichaceae may convert acetyl-CoA to butyryl-CoA via crotonyl-CoA. This pathway, in turn, leads to the production of butyrate^64,65^.

Intestinal microbiota trophic architecture would preserve its ability to produce SCFAs in HIV-infected group. For example, *Prevotella* has a high capacity to degrade fiber, whose products include succinate and acetyl-CoA^66,67^; while, bacteria of the genus *Phascolarctobacterium* can use succinate as a substrate to produce propionate in the intestine^68^. Interestingly, Ling *et al*. found an increase in both *Phascolarctobacterium* and *Prevotella* in the fecal samples of HIV-infected individuals from China^27^. Ling *et al*.^27^ also reported an increase in *Faecalibacterium* and *Butyricicoccus*, which may produce butyric acid^69,70^.

SCFAs may not be so beneficial after all in HIV-infected individuals. Due to histone deacetylases inhibition, SCFAs can activate the transcription and production of viral particles from HIV-1 episomes^71^, reactivate the expression of the provirus integrated in the genome^72,73^, and improve the efficiency of post-entry viral events^74^. Furthermore, even the beneficial effect of inducing Foxp3(+) regulatory T cells^75^ could be detrimental in the context of HIV infection, as this could decrease the response against infected cells, and thereby contribute to the persistence of the virus^73–79^.

Our study has the typical limitations of both an observational study with a cross-sectional design and case-control analyses. First of all, the cross-sectional design of this study only allows us to establish associations without being able to know the exact causal relationship between microbiota signature and immune deregulation. Likewise, the cross-sectional design does not allow us to define the gut microbiota evolutionary dynamics. Although longitudinal studies are desirable, ethical issues place a heavy restriction on conducting such design. The patient is recommended to initiate treatment as soon as a diagnosis is made. However, several studies have reported that the core ecosystem is stable in intervals of 1 year from the first sampling^80–83^. Taking the time it took for patients to be placed under treatment and the stability of the gut microbiota into consideration, a longitudinal study design conducted in a short time interval would not change our major findings. Secondly, fecal microbiota is only a proxy for the microbial community associated with the intestinal mucosa. As such, it is implicit to conduct further studies probing the intestinal mucosa adhered microbial community. Third, we did not directly assess microbial translocation. Fourth, this study did not analyze the changes associated with highly active antiretroviral therapy (HAART). Although it may be argued that a paired post-ART analysis would help in assessing HIV impact on gut microbiota clearly, the major problem is that some of the antiretroviral drugs have an impact on gut microbiota by themselves as each patient is usually subjected to a different treatment scheme based on HIV strain mutational profile that is infecting^84–86^. Since no individuals belonging to a control group is taking ART, it will be very difficult to assign microbiota modifications to viral load, immune changes, or the ART drug itself. Fifth, sample size might be a limitation in our study. Nonetheless, our findings are important to share with the scientific community as they highlight how HIV infection has a differential impact on the intestinal microbiota of a South American community, compared to that reported in studies conducted in developed Western societies.

Certain limitations and pitfalls of massively parallel 16S rRNA gene sequencing need to keep in mind. In contrast to 16S analyses, Whole Metagenome Sequencing (WMS) enables the analysis of the microbial phylogenetic composition and functional diversity^87^. WMS may detect microheterogeneity and genetic intraspecies variations, while bypassing the introduction of additional biases during the PCR amplification steps required in 16S rRNA gene analysis^87,88^. In addition, the high similarity of 16S rRNA amplicon sequences impairs the taxonomic resolution^89^.

Altogether, we have detected the presence of a bacterial signature that can differentiate HIV-infected individuals from non-infected individuals. We call into attention that a community previously overlooked shows a different profile in terms of the impact of HIV infection on gut microbiota. This signature is characterized by an increase in bacteria belonging to the Lachnospiraceae-OTU69, *Blautia*, *Dorea* and Erysipelotrichaceae-OTU109 genera. The Lachnospiraceae-based signature, in the light of previous reports, suggest the need for experimental studies probing intestinal microbiota mechanisms on the context of HIV infection.

## Methods

### Inclusion and exclusion criteria

This study was approved by the ethics committee of the Universidad del Norte. We recruited 25 patients infected with HIV and 12 control individuals. All individuals provided informed consent to participate in the study. HIV-infected patients and control individuals were recruited using the framework and surveys associated to Human Microbiome Project^90,91^. We surveyed both HIV-infected patients and control individuals for demographic data covering socio-economic status, for systems reviews and life-styles (alcohol use and smoking), for drug and antibiotic use, and for diet composition in their meals. In addition, a research team-associated physician conducted a physical examination in each individual. The methods were carried out in accordance with the relevant guidelines and regulations. No patient was undergoing antiretroviral therapy, presented comorbidity with infectious agents such as the hepatitis B and C virus, was experiencing diarrhea at the time of fecal sampling, or had taken antibiotics within the previous 30 days.

We initially recruited all the HIV-infected patients and then control individuals were subsequently recruited based on the combined demographic information of the HIV-infected patients. Demographic information (i.e., age, male/female ratio, socio-economic status, body mass index, and diet composition) was used for matching purposes.

### Stool sample collection

Each individual was asked for a stool sample. Individuals were given a sterile stool-collection recipient to deposit the sample without any contamination from urine. Stool samples while were transported from the collection site to the Universidad del Norte were kept at 4 °C, where they were aliquoted and stored at −150 °C.

### Blood sample collection

For each individual (patients and controls), 30 mL of whole blood was taken, and 20 mL added to a tube containing EDTA and the remaining 10 mL in an empty tube without anticoagulant. Part of the blood that was mixed with EDTA was used to determine the activation state of the CD4 and CD8 cells, while the remainder was centrifuged at 2,500 rpm for 10 min to separate the plasma from the cells. The cell fraction was cultured to determine the percentage of Th17 versus T regulatory lymphocytes.

### Absolute CD4 and CD8 cell counts, CD4/CD8 ratio and percent activation of T cells

Blood was incubated with anti-CD3, -CD4, -CD38, and -HLA-DR antibodies for 10 min using the kit Multitest CD4 FITC/CD38PE/CD3PerCP/anti-HLA-DR-APC (BD Biosciences). Anti-CD8-APC-Cy7 antibody was added to the test (BD Biosciences). BD FACS lysis solution was used to eliminate the red blood cells and to fix the lymphocytes. Absolute CD4 and CD8 cell quantification was carried out using Trucount tubes (BD Biosciences). Cells were processed through BD FACSCANTO II flow cytometer and the BD FACS DIVA software (BD Biosciences). CD4/CD8 ratio was calculated from the respective absolute counts. The percentage of CD3(+)/CD4(+) and CD3(+)/CD8(+) T cells simultaneously expressing HLA-DR(+) and CD38(+) was used to identify activated Th and Tc lymphocytes.

### Lymphocyte cell culture and percentage of Th17 versus regulatory T cells

Peripheral blood mononuclear cells (PBMC) was recovered following Ficoll-Hypaque separation approach. Cells at a concentration of 1 × 10^6^/mL were cultured in 100-mm dishes for 5 h in RPMI medium supplemented with 10% fetal bovine serum, 10 U/mL penicillin-streptomycin, L-glutamine, 20 mM HEPES. T cells were stimulated with PMA and Ionomycin. Golgi Stop was used to accumulate IL17 inside cells. Cells were pelleted, PFA-fixed, and saponin-permeabilized. Cells were labelled with anti-IL-17, anti-FoxP3, anti-CD3, and anti-CD4 antibodies (BD Biosciences Human Th17/Treg Phenotyping kit). Th17 and Treg (Foxp3+) cells percentages were determined using the BD FACSCANTO II flow cytometer (BD Bioscience) and the BD FacsDiva software (BD Biosciences).

### DNA extraction, 16S rRNA gene sequencing and bioinformatics analysis

16S rRNA gene compositional analysis provides a summary of the composition and structure of the bacterial component of the microbiome. Genomic bacterial DNA extraction methods were optimized to maximize the yield of bacterial DNA while keeping background amplification to a minimum. 16S rRNA gene sequencing methods were adapted from the methods developed for the NIH-Human Microbiome Project^90,91^. Briefly, bacterial genomic DNA was extracted using MO BIO PowerSoil DNA Isolation Kit (MO BIO Laboratories). The 16S rDNA V4 region was amplified by PCR and sequenced in the MiSeq platform (Illumina) using the 2 × 250 bp paired-end protocol yielding pair-end reads that overlap almost completely. The primers used for amplification contain adapters for MiSeq sequencing and dual-index barcodes so that the PCR products may be pooled and sequenced directly^92^, targeting at least 10,000 reads per sample.

Our standard pipeline for processing and analyzing the 16S rRNA gene data incorporated phylogenetic and alignment-based approaches to maximize data resolution. The read pairs were demultiplexed based on the unique molecular barcodes, and reads were merged using USEARCH v7.0.1001^93^ allowing zero mismatches and a minimum overlap of 50 bases. Merged reads were trimmed at first base with Q5. In addition, a quality filter was applied to the resulting merged reads and reads containing above 0.05 expected errors were discarded.

We used an in-house pipeline for 16S analysis as well as several other tools, including custom analytic packages developed at the CMMR to provide summary statistics and quality control measurements for each sequencing run, as well as multi-run reports and data-merging capabilities for validating built-in controls and characterizing microbial communities across large numbers of samples or sample groups.

16S rRNA gene sequences were assigned into Operational Taxonomic Units (OTUs) or phylotypes at a similarity cutoff value of 97% using the UPARSE algorithm. OTUs are then mapped to an optimized version (v.111) of the SILVA Database^94,95^ containing only the 16S v4 region to determine taxonomies Abundances were recovered by mapping the demultiplexed reads to the UPARSE OTUs. A custom script constructs an OTU table from the output files generated in the previous two steps, which is then used to calculate α-diversity, β-diversity^96^, and provide taxonomic summaries that were leveraged for all subsequent analyses discussed below.

### Data analysis

In order to construct DOC curves, the dissimilarity and overlapping measure must first be defined. Here, we used the measures proposed by Bashan *et al*.^32^. The measures were then plotted as points in a scatterplot. Subsequently, the DOC area was calculated from LOWESS method. The cutoff point, *O*_*c*_, from which the slope of the curve is always negative is then calculated (i.e., $\frac{\partial D}{\partial O} < 0|O > {O}_{c}$). The fraction *f*_*ns*_ is calculated as follows:

$${f}_{ns}=\frac{{number}\,{of}\,{sample}\,{pairs}\,{with}\,{O} > {{O}}_{{c}}}{{total}\,{number}\,{of}\,{sample}\,{pairs}}$$

To calculate the p-values related to the DOC plot slope we carry out the procedure described in the paper published by Bashan *et al*.^32^. We retain only data points showing an overlap value greater than the median for both the control and disease groups. A bootstrap procedure repeating the aforementioned steps is performed and the slopes of the DOC plots are calculated at every bootstrap realization. The *P*-values are finally calculated as the fraction of bootstrap realizations resulting with non-negative slopes. The number of bootstrap runs was named n_boot.

The compositional nature of the data restricts the data analysis to a simplex space. As such, we applied the Aitchison’s centered log ratio transformation (CLR) to carry the data to a Euclidean space. In this way, each OTU is properly compared between the different samples^34,35^. The logratio.transfo function of the mixOmics package was used for CLR transformation^97,98^. A multiplicative Bayesian replacement strategy using the cmultRepl function of the zCompositions package allowed us to replace zeros^99^.

We performed a Jennrich test^38^ to calculate the p-value associated to the correlation structures between HIV-infected patients control group.

SPIEC-EASI^39^ was employed using the strategy of Meinshausen-Buhlmann for graph estimation of the network, which is a modified precision matrix built from β coefficients calculated from the average nearest neighbor. To reduce the number of false positive during the construction of networks, we eliminated from the analysis any OTU that was not present in at least 50% of the samples^100^. sPLS-DA and Random Forest were techniques used for feature selection. sPLS-DA used an approach that asks and identifies which features (OTUs) separate the HIV-infected group from control group based on a discriminant analysis of the partial least square metric^40^. Random Forest^101^, on the other hand, is a machine learning technique that also identifies features (OTUs) based on a construction of collection of decision trees with controlled variance^102^. Variables capable of differentiating between the groups were verified by Sparse PLS discriminant analysis (sPLS-DA)^40^. The most important bacteria were grouped in the first component. The Random Forest technique was also applied in order to determine the importance of bacteria in marking differences between the different patient groups. The performance of bacteria was measured using clr-normalized data and taking into account the mean decrease Gini^78,79^. To analyze the differences in microbial communities between the distinct patient groups, we used metagenomeSeq. For this, data was normalized by cumulative sum scaling using the cumNorm function, and analysis was performed with the zero-inflated log-normal fitZigModel function^44^.

### Data availability

The sequence data from this study are deposited in the Bioproject database (BioProject ID is PRJNA408085).

## Electronic supplementary material

Supplementary Information
