# Supplementary material for: A Lachnospiraceae-dominated bacterial signature in the fecal microbiota of HIV-infected individuals from Colombia, South America
Source: Sci Rep. 2018 Mar 14;8:4479. doi: 10.1038/s41598-018-22629-7 (PMC5852036; doi:10.1038/s41598-018-22629-7)
Supplement: Supplementary file 1 — Supplementary Information [file 41598_2018_22629_MOESM1_ESM.pdf]

SUPPLEMENTARY INFORMATION

A Lachnospiraceae-dominated bacterial signature in the fecal microbiota of HIV-infected individuals from Colombia, South America.

Homero San-Juan-Vergara*[1], Eduardo Zurek[2], Nadim J. Ajami[3], Christian Mogollon[4], Mario Peña[1], Ivan Portnoy[2], Jorge I. Velez[2], Christian Cadena-Cruz[1], Yirys Diaz-Olmos[1], Leidy Hurtado-Gómez[1], Silvana Sanchez-Sit[5], Danitza Hernández[4], Irina Urruchurtu[4], Pierina Di-Ruggiero[4], Ella Guardo-García[6], Nury Torres[6], Oscar Vidal-Orjuela[1], Diego Viasus[1], Joseph F. Petrosino[3], Guillermo Cervantes-Acosta*[1].

[1.] División Ciencias de la Salud. Fundación Universidad del Norte, Colombia

[2.] División de Ingenierías. Fundación Universidad del Norte, Colombia

[3.] Alkek Center for Metagenomics and Microbiome Research, Department of Molecular Virology and Microbiology, Baylor College of Medicine. Houston, Texas, USA

[4.] IPS Medicina Integral, Colombia

[5]. Maestría en Estadística Aplicada. Universidad del Norte, Colombia.

[6.] IPS de la Costa, Colombia

* Homero Gabriel San-Juan-Vergara, Professor Department of Medicine. Division of Health Sciences. Universidad del Norte. E-mail: hsanjuan@uninorte.edu.co

* Guillermo Cervantes-Acosta, Professor Department of Medicine. Division of Health Sciences. Universidad del Norte. E-mail: guicerva@uninorte.edu.co

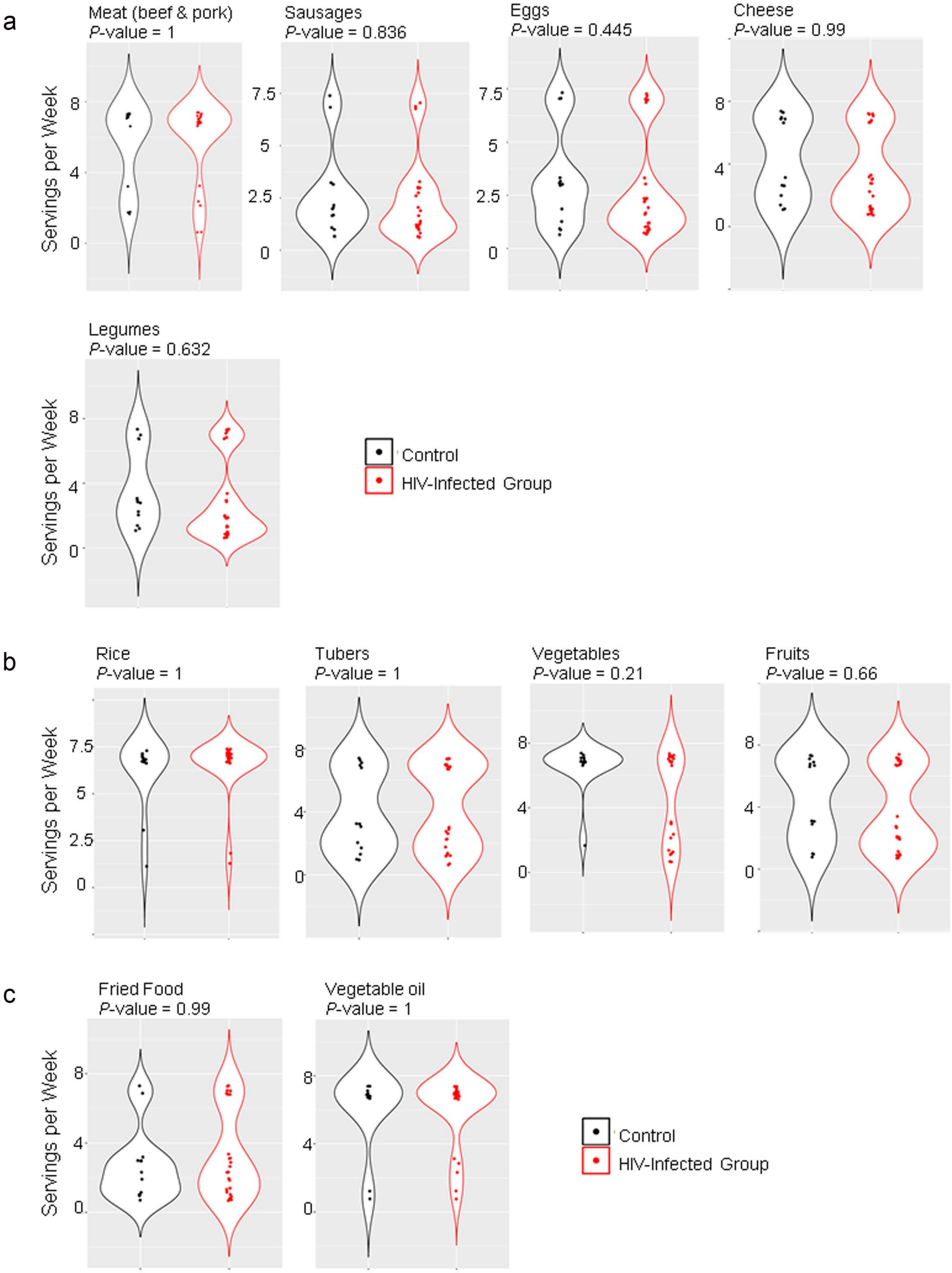

Supplementary Figure S1. Similar diet composition between control individuals and HIV-infected group. (a) Protein consumption in servings per week. (b) Carbohydrates consumption in servings per week. c) Fat consumption in servings per week. *P*-value was calculated using two-sample Kolmogorov-Smirnov test.

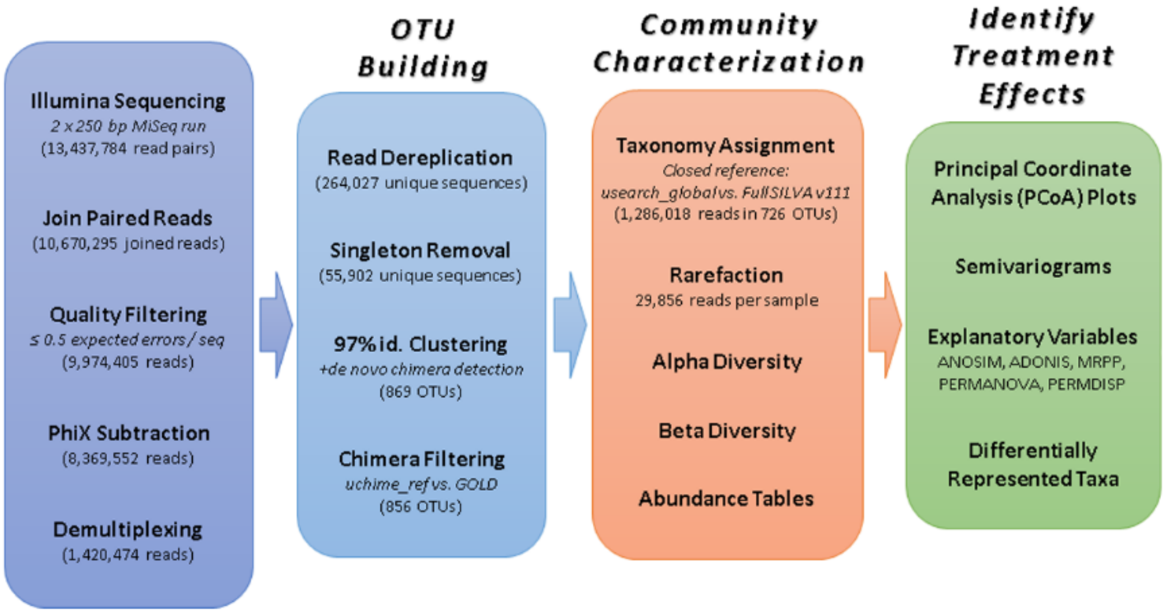

Supplementary Figure S2. Workflow for assigning taxonomies and constructing abundance tables.

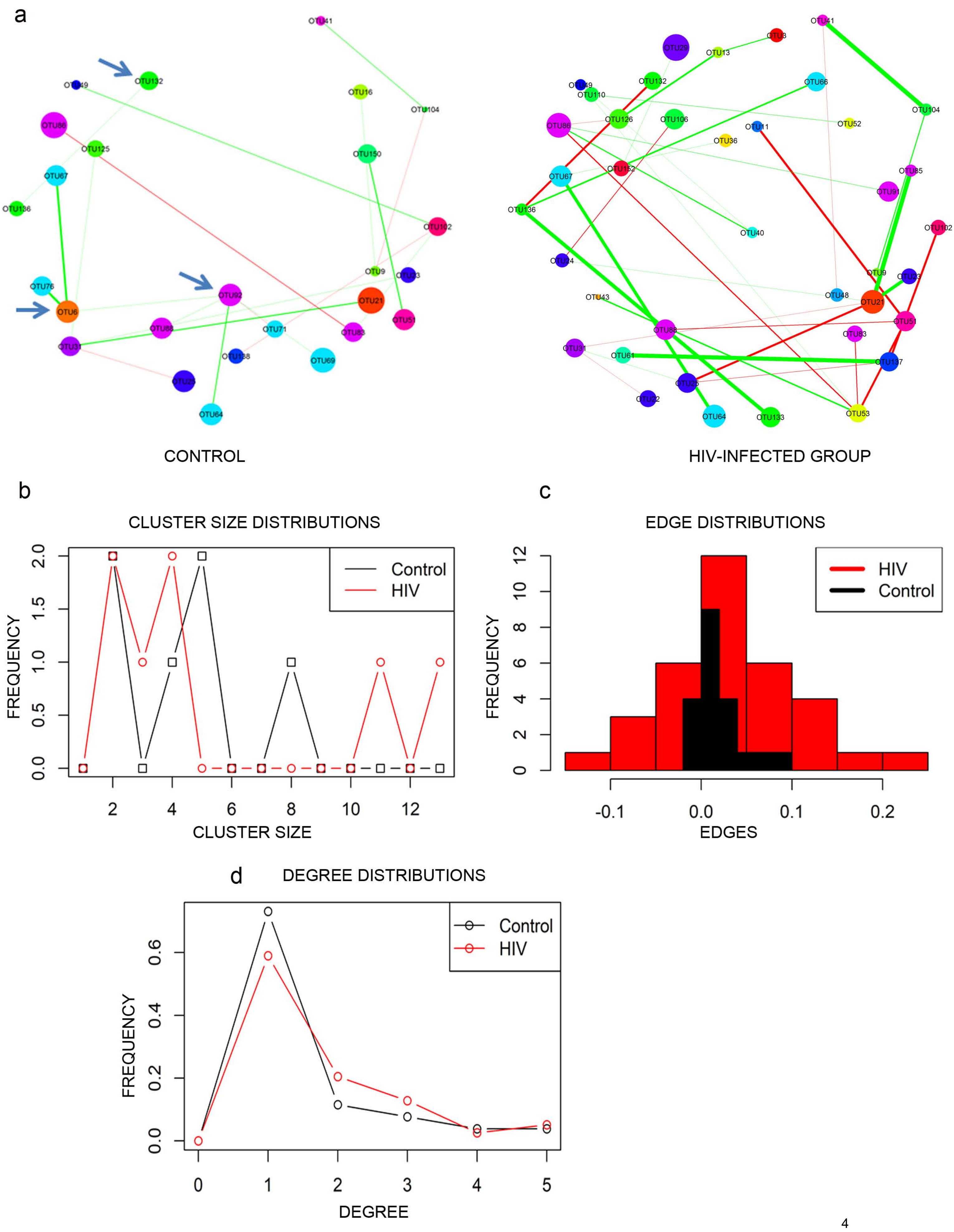
a
OTU41
OTU132
OTU16
OTU104
OTU86
OTU125
OTU150
OTU67
OTU136
OTU102
OTU9
OTU76
OTU92
OTU21
OTU6
OTU88
OTU71
OTU51
OTU83
OTU31
OTU69
OTU64
CONTROL
OTU29
OTU13
OTU3
OTU66
OTU49
OTU110
OTU126
OTU106
OTU11
OTU52
OTU36
OTU152
OTU85
OTU91
OTU40
OTU24
OTU43
OTU48
OTU61
OTU137
OTU53
OTU133
OTU22
HIV-INFECTED GROUP
b
CLUSTER SIZE DISTRIBUTIONS
Control
HIV
FREQUENCY
0.0
0.5
1.0
1.5
2.0
2
4
6
8
10
12
CLUSTER SIZE
c
EDGE DISTRIBUTIONS
HIV
Control
FREQUENCY
0
2
4
6
8
10
12
-0.1
0.0
0.1
0.2
EDGES
d
DEGREE DISTRIBUTIONS
Control
HIV
FREQUENCY
0.0
0.2
0.4
0.6
0
1
2
3
4
5
DEGREE

Supplementary Figure S3. Networks structure and analysis. (a) Networks whose nodes were fixed in their positions. Arrows indicate those OTUs that dissapear in HIV-infected network. (b) Cluster size distributions. (c) Edge distibutions. (d) Degree distributions

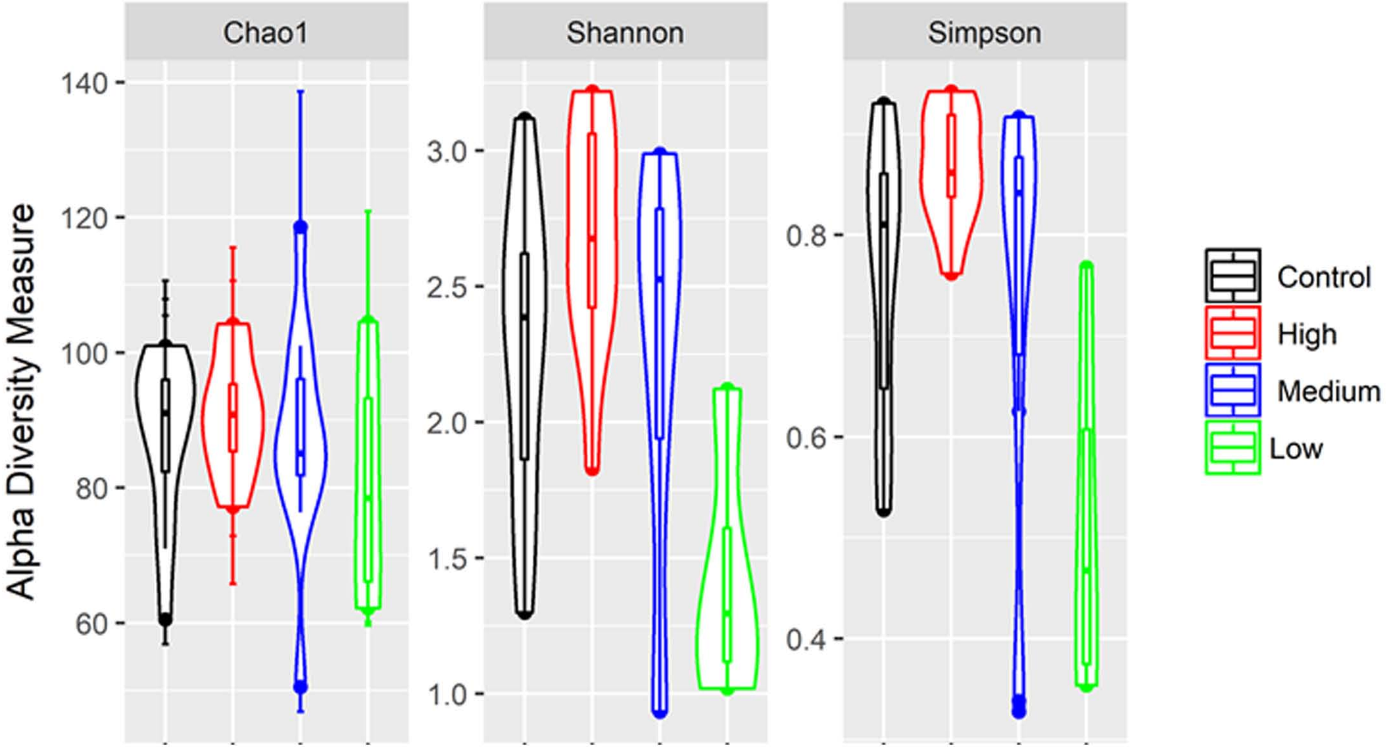

High vs. Control. *P*-value = 0.069
High vs Low *P*-value = 0.0039

High vs. Control. *P*-value = 0.059
High vs Low *P*-value = 0.0039

Supplementary Figure S4. Comparison of alpha-diversity indexes among subgroups. P-value was calculated using Mann-Whitney-Wilcoxon test.

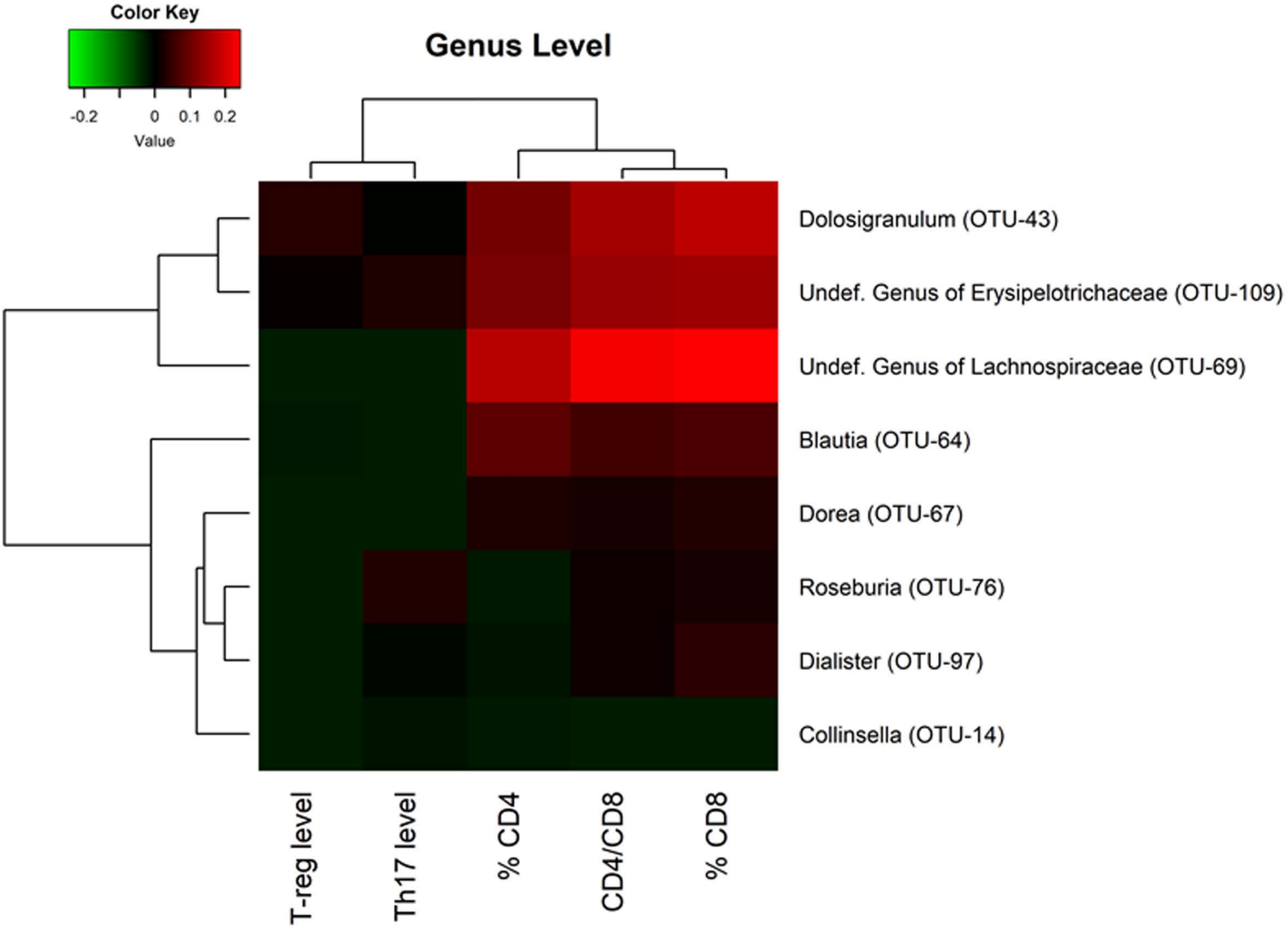

Supplementary Figure S5. Clustergram showing correlations of immune parameters with OTUs identified as the features able to separate Control group and HIV-infected group.

Supplementary Table S1. Alpha diversity (Observed Species, Chao Index, Shannon Index) for each of the fecal samples.

SAMPLE ID	Observed Species	Chao	Shannon
C.27	762	1045	6.81
V.10	738	1003	6.52
V.26	724	1022	6.48
C.10	693	988	6.49
V.02	661	956	6.20
C.26	654	910	6.44
V.24	653	893	6.53
V.17	641	959	5.75
V.16	617	828	5.84
V.06	614	786	6.27
V.09	613	787	6.01
V.13	596	872	5.52
V.19	573	810	6.04
V.12	570	753	5.75
V.11	549	730	6.07
C.05	528	722	5.01
V.01	514	747	4.81
V.14	509	809	4.57
V.18	502	740	5.26
V.05	499	742	5.03
V.15	496	708	5.48
C.14	495	708	4.86
C.01	479	735	4.67
C.02	474	692	5.41
V.23	441	590	5.26
C.24	437	611	5.46
V.04	427	614	4.72
V.08	412	615	4.21
C.23	386	557	4.68
C.25	350	481	4.63
C.09	342	566	4.42
V.20	304	505	3.68

SAMPLE ID	Observed Species	Chao	Shannon
V.25	283	429	4.14
V.27	274	451	3.82
V.07	255	329	3.84
V.03	222	368	3.47
C.04	182	261	3.72

Supplementary Table S2. Code-labeling for each OTU / Taxon

Taxon	CODE
Archaea;__Euryarchaeota;__Methanobacteria;__Methanobacteriales;__Methanobacteriaceae;__Methanobrevibacter	1
Archaea;__Euryarchaeota;__Methanobacteria;__Methanobacteriales;__Methanobacteriaceae;__Methanosphaera	2
Bacteria;__Actinobacteria;__Actinobacteria;__Actinomycetales;__Actinomycetaceae;__Actinomyces	3
Bacteria;__Actinobacteria;__Actinobacteria;__Actinomycetales;__Actinomycetaceae;__Varibaculum	4
Bacteria;__Actinobacteria;__Actinobacteria;__Bifidobacteriales;__Bifidobacteriaceae;__Alloscardovia	5
Bacteria;__Actinobacteria;__Actinobacteria;__Bifidobacteriales;__Bifidobacteriaceae;__Bifidobacterium	6
Bacteria;__Actinobacteria;__Actinobacteria;__Bifidobacteriales;__Bifidobacteriaceae;__Gardnerella	7
Bacteria;__Actinobacteria;__Actinobacteria;__Bifidobacteriales;__Bifidobacteriaceae;__Scardovia	8
Bacteria;__Actinobacteria;__Actinobacteria;__Corynebacteriales;__Corynebacteriaceae;__Corynebacterium	9
Bacteria;__Actinobacteria;__Actinobacteria;__Micrococcales;__Micrococcaceae;__Kocuria	10
Bacteria;__Actinobacteria;__Actinobacteria;__Micrococcales;__Micrococcaceae;__Rothia	11
Bacteria;__Actinobacteria;__Coriobacteriia;__Coriobacteriales;__Coriobacteriaceae;__Adlercreutzia	12
Bacteria;__Actinobacteria;__Coriobacteriia;__Coriobacteriales;__Coriobacteriaceae;__Atopobium	13
Bacteria;__Actinobacteria;__Coriobacteriia;__Coriobacteriales;__Coriobacteriaceae;__Collinsella	14
Bacteria;__Actinobacteria;__Coriobacteriia;__Coriobacteriales;__Coriobacteriaceae;__Eggerthella	15
Bacteria;__Actinobacteria;__Coriobacteriia;__Coriobacteriales;__Coriobacteriaceae;__Enterorhabdus	16
Bacteria;__Actinobacteria;__Coriobacteriia;__Coriobacteriales;__Coriobacteriaceae;__g	17
Bacteria;__Actinobacteria;__Coriobacteriia;__Coriobacteriales;__Coriobacteriaceae;__Gordonibacter	18
Bacteria;__Actinobacteria;__Coriobacteriia;__Coriobacteriales;__Coriobacteriaceae;__Olsenella	19
Bacteria;__Actinobacteria;__Coriobacteriia;__Coriobacteriales;__Coriobacteriaceae;__Slackia	20
Bacteria;__Bacteroidetes;__Bacteroidia;__Bacteroidales;__Bacteroidaceae;__Bacteroides	21
Bacteria;__Bacteroidetes;__Bacteroidia;__Bacteroidales;__Porphyromonadaceae;__Barnesiella	22
Bacteria;__Bacteroidetes;__Bacteroidia;__Bacteroidales;__Porphyromonadaceae;__Butyricimonas	23
Bacteria;__Bacteroidetes;__Bacteroidia;__Bacteroidales;__Porphyromonadaceae;__Odoribacter	24
Bacteria;__Bacteroidetes;__Bacteroidia;__Bacteroidales;__Porphyromonadaceae;__Parabacteroides	25
Bacteria;__Bacteroidetes;__Bacteroidia;__Bacteroidales;__Porphyromonadaceae;__Porphyromonas	26
Bacteria;__Bacteroidetes;__Bacteroidia;__Bacteroidales;__Prevotellaceae;__g	27

Taxon	CODE
Bacteria;__Bacteroidetes;__Bacteroidia;__Bacteroidales;__Prevotellaceae;__Paraprevotella	28
Bacteria;__Bacteroidetes;__Bacteroidia;__Bacteroidales;__Prevotellaceae;__Prevotella	29
Bacteria;__Bacteroidetes;__Bacteroidia;__Bacteroidales;__RF16;__g	30
Bacteria;__Bacteroidetes;__Bacteroidia;__Bacteroidales;__Rikenellaceae;__Alistipes	31
Bacteria;__Bacteroidetes;__Bacteroidia;__Bacteroidales;__Rikenellaceae;__RC9_gut_group	32
Bacteria;__Bacteroidetes;__Bacteroidia;__Bacteroidales;__S24-7;__g	33
Bacteria;__Bacteroidetes;__Bacteroidia;__Bacteroidales;__uncultured;__g	34
Bacteria;__Cyanobacteria;__4C0d-2;__o;__f;__g	35
Bacteria;__Cyanobacteria;__Chloroplast;__Chloroplast;__Chloroplast;__Chloroplast	36
Bacteria;__Elusimicrobia;__Elusimicrobia;__Elusimicrobiales;__Elusimicrobiaceae;__Elusimicrobium	37
Bacteria;__Firmicutes;__Bacilli;__Bacillales;__Bacillaceae;__Bacillus	38
Bacteria;__Firmicutes;__Bacilli;__Bacillales;__Bacillaceae;__Geobacillus	39
Bacteria;__Firmicutes;__Bacilli;__Bacillales;__Family_XI_Incertae_Sedis;__Gemella	40
Bacteria;__Firmicutes;__Bacilli;__Bacillales;__Staphylococcaceae;__Staphylococcus	41
Bacteria;__Firmicutes;__Bacilli;__Lactobacillales;__Aerococcaceae;__Abiotrophia	42
Bacteria;__Firmicutes;__Bacilli;__Lactobacillales;__Carnobacteriaceae;__Dolosigranulum	43
Bacteria;__Firmicutes;__Bacilli;__Lactobacillales;__Enterococcaceae;__Enterococcus	44
Bacteria;__Firmicutes;__Bacilli;__Lactobacillales;__Lactobacillaceae;__Lactobacillus	45
Bacteria;__Firmicutes;__Bacilli;__Lactobacillales;__Leuconostocaceae;__Fructobacillus	46
Bacteria;__Firmicutes;__Bacilli;__Lactobacillales;__Leuconostocaceae;__Leuconostoc	47
Bacteria;__Firmicutes;__Bacilli;__Lactobacillales;__Leuconostocaceae;__Weissella	48
Bacteria;__Firmicutes;__Bacilli;__Lactobacillales;__PeH08;__g	49
Bacteria;__Firmicutes;__Bacilli;__Lactobacillales;__Streptococcaceae;__Lactococcus	50
Bacteria;__Firmicutes;__Bacilli;__Lactobacillales;__Streptococcaceae;__Streptococcus	51
Bacteria;__Firmicutes;__Clostridia;__Clostridiales;__Christensenellaceae;__Christensenella	52
Bacteria;__Firmicutes;__Clostridia;__Clostridiales;__Christensenellaceae;__g	53
Bacteria;__Firmicutes;__Clostridia;__Clostridiales;__Clostridiaceae;__Clostridium	54

Taxon	CODE
Bacteria;__Firmicutes;__Clostridia;__Clostridiales;__Clostridiaceae;__Sarcina	55
Bacteria;__Firmicutes;__Clostridia;__Clostridiales;__Eubacteriaceae;__Eubacterium	56
Bacteria;__Firmicutes;__Clostridia;__Clostridiales;__Family_XI_Incertae_Sedis;__Anaerococcus	57
Bacteria;__Firmicutes;__Clostridia;__Clostridiales;__Family_XI_Incertae_Sedis;__Finegoldia	58
Bacteria;__Firmicutes;__Clostridia;__Clostridiales;__Family_XI_Incertae_Sedis;__Parvimonas	59
Bacteria;__Firmicutes;__Clostridia;__Clostridiales;__Family_XI_Incertae_Sedis;__Peptoniphilus	60
Bacteria;__Firmicutes;__Clostridia;__Clostridiales;__Family_XIII_Incertae_Sedis;__Incertae_Sedis	61
Bacteria;__Firmicutes;__Clostridia;__Clostridiales;__Family_XIII_Incertae_Sedis;__Mogibacterium	62
Bacteria;__Firmicutes;__Clostridia;__Clostridiales;__Lachnospiraceae;__Anaerostipes	63
Bacteria;__Firmicutes;__Clostridia;__Clostridiales;__Lachnospiraceae;__Blautia	64
Bacteria;__Firmicutes;__Clostridia;__Clostridiales;__Lachnospiraceae;__Butyrivibrio	65
Bacteria;__Firmicutes;__Clostridia;__Clostridiales;__Lachnospiraceae;__Coprococcus	66
Bacteria;__Firmicutes;__Clostridia;__Clostridiales;__Lachnospiraceae;__Dorea	67
Bacteria;__Firmicutes;__Clostridia;__Clostridiales;__Lachnospiraceae;__Howardella	68
Bacteria;__Firmicutes;__Clostridia;__Clostridiales;__Lachnospiraceae;__Incertae_Sedis	69
Bacteria;__Firmicutes;__Clostridia;__Clostridiales;__Lachnospiraceae;__Johnsonella	70
Bacteria;__Firmicutes;__Clostridia;__Clostridiales;__Lachnospiraceae;__Lachnospira	71
Bacteria;__Firmicutes;__Clostridia;__Clostridiales;__Lachnospiraceae;__Marvinbryantia	72
Bacteria;__Firmicutes;__Clostridia;__Clostridiales;__Lachnospiraceae;__Moryella	73
Bacteria;__Firmicutes;__Clostridia;__Clostridiales;__Lachnospiraceae;__Oribacterium	74
Bacteria;__Firmicutes;__Clostridia;__Clostridiales;__Lachnospiraceae;__Pseudobutyrivibrio	75
Bacteria;__Firmicutes;__Clostridia;__Clostridiales;__Lachnospiraceae;__Roseburia	76
Bacteria;__Firmicutes;__Clostridia;__Clostridiales;__Lachnospiraceae;__Shuttleworthia	77
Bacteria;__Firmicutes;__Clostridia;__Clostridiales;__Peptococcaceae;__g	78
Bacteria;__Firmicutes;__Clostridia;__Clostridiales;__Peptococcaceae;__Peptococcus	79

Taxon	CODE
Bacteria;__Firmicutes;__Clostridia;__Clostridiales;__Peptostreptococcaceae;__Incertae_Sedis	80
Bacteria;__Firmicutes;__Clostridia;__Clostridiales;__Peptostreptococcaceae;__Peptostreptococcus	81
Bacteria;__Firmicutes;__Clostridia;__Clostridiales;__Ruminococcaceae;__Acetanaerobacterium	82
Bacteria;__Firmicutes;__Clostridia;__Clostridiales;__Ruminococcaceae;__Anaerotruncus	83
Bacteria;__Firmicutes;__Clostridia;__Clostridiales;__Ruminococcaceae;__Faecalibacterium	84
Bacteria;__Firmicutes;__Clostridia;__Clostridiales;__Ruminococcaceae;__Flavonifractor	85
Bacteria;__Firmicutes;__Clostridia;__Clostridiales;__Ruminococcaceae;__g	86
Bacteria;__Firmicutes;__Clostridia;__Clostridiales;__Ruminococcaceae;__Hydrogenoanaerobacterium	87
Bacteria;__Firmicutes;__Clostridia;__Clostridiales;__Ruminococcaceae;__Incertae_Sedis	88
Bacteria;__Firmicutes;__Clostridia;__Clostridiales;__Ruminococcaceae;__Oscillibacter	89
Bacteria;__Firmicutes;__Clostridia;__Clostridiales;__Ruminococcaceae;__Oscillospira	90
Bacteria;__Firmicutes;__Clostridia;__Clostridiales;__Ruminococcaceae;__Ruminococcus	91
Bacteria;__Firmicutes;__Clostridia;__Clostridiales;__Ruminococcaceae;__Subdoligranulum	92
Bacteria;__Firmicutes;__Clostridia;__Clostridiales;__uncultured;__g	93
Bacteria;__Firmicutes;__Clostridia;__Clostridiales;__Veillonellaceae;__Acidaminococcus	94
Bacteria;__Firmicutes;__Clostridia;__Clostridiales;__Veillonellaceae;__Allisonella	95
Bacteria;__Firmicutes;__Clostridia;__Clostridiales;__Veillonellaceae;__Anaerovibrio	96
Bacteria;__Firmicutes;__Clostridia;__Clostridiales;__Veillonellaceae;__Dialister	97
Bacteria;__Firmicutes;__Clostridia;__Clostridiales;__Veillonellaceae;__Megamonas	98
Bacteria;__Firmicutes;__Clostridia;__Clostridiales;__Veillonellaceae;__Megasphaera	99
Bacteria;__Firmicutes;__Clostridia;__Clostridiales;__Veillonellaceae;__Mitsuokella	100
Bacteria;__Firmicutes;__Clostridia;__Clostridiales;__Veillonellaceae;__Phascolarctobacterium	101
Bacteria;__Firmicutes;__Clostridia;__Clostridiales;__Veillonellaceae;__Veillonella	102
Bacteria;__Firmicutes;__Clostridia;__Thermoanaerobacterales;__Thermoanaerobacteraceae;__Gelria	103
Bacteria;__Firmicutes;__Erysipelotrichi;__Erysipelotrichales;__Erysipelotrichaceae;__Allobaculum	104
Bacteria;__Firmicutes;__Erysipelotrichi;__Erysipelotrichales;__Erysipelotrichaceae;__Asteroleplasma	105
Bacteria;__Firmicutes;__Erysipelotrichi;__Erysipelotrichales;__Erysipelotrichaceae;__Catenibacterium	106

Taxon	CODE
Bacteria;__Firmicutes;__Erysipelotrichi;__Erysipelotrichales;__Erysipelotrichaceae;__g	107
Bacteria;__Firmicutes;__Erysipelotrichi;__Erysipelotrichales;__Erysipelotrichaceae;__Holdemania	108
Bacteria;__Firmicutes;__Erysipelotrichi;__Erysipelotrichales;__Erysipelotrichaceae;__Incertae_Sedis	109
Bacteria;__Firmicutes;__Erysipelotrichi;__Erysipelotrichales;__Erysipelotrichaceae;__Solobacterium	110
Bacteria;__Firmicutes;__Erysipelotrichi;__Erysipelotrichales;__Erysipelotrichaceae;__Turicibacter	111
Bacteria;__Fusobacteria;__Fusobacteria;__Fusobacteriales;__Fusobacteriaceae;__Cetobacterium	112
Bacteria;__Fusobacteria;__Fusobacteria;__Fusobacteriales;__Fusobacteriaceae;__Fusobacterium	113
Bacteria;__Fusobacteria;__Fusobacteria;__Fusobacteriales;__Leptotrichiaceae;__Leptotrichia	114
Bacteria;__Fusobacteria;__Fusobacteria;__Fusobacteriales;__Leptotrichiaceae;__Sneathia	115
Bacteria;__Lentisphaerae;__Lentisphaeria;__Victivallales;__Victivallaceae;__Victivallis	116
Bacteria;__Proteobacteria;__Alphaproteobacteria;__Rhodospirillales;__Acetobacteraceae;__Gluconobacter	117
Bacteria;__Proteobacteria;__Alphaproteobacteria;__Rhodospirillales;__Rhodospirillaceae;__Thalassospira	118
Bacteria;__Proteobacteria;__Alphaproteobacteria;__Rickettsiales;__mitochondria;__g	119
Bacteria;__Proteobacteria;__Betaproteobacteria;__Burkholderiales;__Alcaligenaceae;__Parasutterella	120
Bacteria;__Proteobacteria;__Betaproteobacteria;__Burkholderiales;__Alcaligenaceae;__Sutterella	121
Bacteria;__Proteobacteria;__Betaproteobacteria;__Burkholderiales;__Comamonadaceae;__Comamonas	122
Bacteria;__Proteobacteria;__Betaproteobacteria;__Burkholderiales;__Oxalobacteraceae;__Oxalobacter	123
Bacteria;__Proteobacteria;__Betaproteobacteria;__Neisseriales;__Neisseriaceae;__Neisseria	124
Bacteria;__Proteobacteria;__Deltaproteobacteria;__Desulfovibrionales;__Desulfovibrionaceae;__Bilophila	125
Bacteria;__Proteobacteria;__Deltaproteobacteria;__Desulfovibrionales;__Desulfovibrionaceae;__Desulfovibrio	126
Bacteria;__Proteobacteria;__Epsilonproteobacteria;__Campylobacterales;__Campylobacteraceae;__Campylobacter	127
Bacteria;__Proteobacteria;__Epsilonproteobacteria;__Campylobacterales;__Helicobacteraceae;__Helicobacter	128
Bacteria;__Proteobacteria;__Gammaproteobacteria;__Aeromonadales;__Succinivibrionaceae;__Anaerobiospirillum	129
Bacteria;__Proteobacteria;__Gammaproteobacteria;__Aeromonadales;__Succinivibrionaceae;__Ruminobacter	130
Bacteria;__Proteobacteria;__Gammaproteobacteria;__Aeromonadales;__Succinivibrionaceae;__Succinivibrio	131
Bacteria;__Proteobacteria;__Gammaproteobacteria;__Enterobacteriales;__Enterobacteriaceae;__Enterobacter	132
Bacteria;__Proteobacteria;__Gammaproteobacteria;__Enterobacteriales;__Enterobacteriaceae;__Escherichia-Shigella	133

Taxon	CODE
Bacteria;__Proteobacteria;__Gammaproteobacteria;__Enterobacteriales;__Enterobacteriaceae;__Proteus	134
Bacteria;__Proteobacteria;__Gammaproteobacteria;__Enterobacteriales;__Enterobacteriaceae;__Providencia	135
Bacteria;__Proteobacteria;__Gammaproteobacteria;__Enterobacteriales;__Enterobacteriaceae;__Yersinia	136
Bacteria;__Proteobacteria;__Gammaproteobacteria;__Pasteurellales;__Pasteurellaceae;__Actinobacillus	137
Bacteria;__Proteobacteria;__Gammaproteobacteria;__Pasteurellales;__Pasteurellaceae;__Haemophilus	138
Bacteria;__Proteobacteria;__Gammaproteobacteria;__Pseudomonadales;__Moraxellaceae;__Acinetobacter	139
Bacteria;__Proteobacteria;__Gammaproteobacteria;__Pseudomonadales;__Moraxellaceae;__Enhydrobacter	140
Bacteria;__Proteobacteria;__Gammaproteobacteria;__Pseudomonadales;__Moraxellaceae;__Moraxella	141
Bacteria;__Proteobacteria;__Gammaproteobacteria;__Pseudomonadales;__Moraxellaceae;__Psychrobacter	142
Bacteria;__Proteobacteria;__Gammaproteobacteria;__Pseudomonadales;__Pseudomonadaceae;__Pseudomonas	143
Bacteria;__Proteobacteria;__Gammaproteobacteria;__Xanthomonadales;__Xanthomonadaceae;__Stenotrophomonas	144
Bacteria;__RF3;__c;__o;__f;__g	145
Bacteria;__Spirochaetes;__Spirochaetes;__Spirochaetales;__Spirochaetaceae;__Treponema	146
Bacteria;__Synergistetes;__Synergistia;__Synergistales;__Synergistaceae;__Cloacibacillus	147
Bacteria;__Tenericutes;__Mollicutes;__Anaeroplasmatales;__Anaeroplasmataceae;__Anaeroplasma	148
Bacteria;__Tenericutes;__Mollicutes;__Mycoplasmatales;__Mycoplasmataceae;__Ureaplasma	149
Bacteria;__Tenericutes;__Mollicutes;__RF9;__f;__g	150
Bacteria;__Verrucomicrobia;__Opitutae;__vadinHA64;__f;__g	151
Bacteria;__Verrucomicrobia;__Verrucomicrobiae;__Verrucomicrobiales;__Verrucomicrobiaceae;__Akkermansia	152

Supplementary Table S3. Bacterial Counts in HIV-infected patients with the highest percentage of activated CD4(+) T cells (>10% HIGH)

CODE	V.01	V.03	V.04	V.06	V.09	V.10	V.15	V.19	V.24	V.26
1	0	0	0	0	23	208	1	0	5	79
2	0	0	0	0	0	0	0	0	0	0
3	6	53	6	36	3	10	4	9	53	25
4	0	0	0	0	0	0	0	0	0	0
5	0	2	0	0	0	0	0	0	0	0
6	164	4	1	19	16	2670	696	73	2589	69
7	0	0	0	0	2	0	0	0	0	0
8	0	1	1	0	0	0	0	0	2	0
9	6	6	4	5	4	9	7	1	11	3
10	0	0	0	0	0	0	0	0	0	0
11	0	9	3	18	0	0	1	0	44	2
12	0	0	0	0	0	15	26	0	0	2
13	2	9	1	3	0	3	0	0	3	5
14	27	1	3	82	92	50	1	80	97	238
15	0	0	0	0	1	9	5	0	0	0
16	9	0	0	41	19	21	0	2	53	25
17	17	0	2	97	70	40	0	27	134	87
18	0	0	0	0	0	0	0	0	0	0
19	0	0	0	0	0	0	0	0	0	0
20	3	0	2	2	26	0	0	5	6	28
21	724	5	1068	273	51	6743	16709	4856	3181	229
22	9	0	428	0	1	1190	2102	0	33	0
23	54	0	70	16	0	51	53	55	0	26
24	60	0	0	108	1	275	165	20	45	5
25	144	1	490	46	75	344	1053	85	2209	44

CODE	V.01	V.03	V.04	V.06	V.09	V.10	V.15	V.19	V.24	V.26
26	0	0	0	253	0	3	0	379	2	0
27	106	2	791	517	291	48	50	259	1266	1457
28	312	0	0	0	0	0	0	0	50	5
29	10564	18	7863	7604	5011	189	6	4221	3947	11651
30	0	0	0	0	0	0	0	0	0	0
31	1158	0	251	143	23	1355	2932	47	548	4
32	388	0	1	4517	791	454	0	200	0	541
33	10	4	11	186	1387	10	2	597	666	192
34	116	0	0	0	403	0	203	58	0	222
35	0	0	0	36	8	0	0	0	76	75
36	7	126	1	2	0	3	0	0	0	2
37	0	0	0	25	0	0	0	2	0	0
38	2	1	1	2	0	1	2	2	4	1
39	0	0	0	0	0	0	0	0	0	0
40	0	14	1	6	0	2	1	39	12	2
41	8	4	17	6	7	14	11	14	12	1
42	0	0	1	1	0	0	0	0	0	0
43	1	0	2	2	3	1	3	7	3	2
44	1	14	1	2	0	2	3	1354	2	0
45	16	3	359	120	0	2	36	47	53	1
46	0	0	0	0	0	0	0	0	0	0
47	0	0	0	0	0	0	0	0	0	0
48	1	0	14	1	0	2	0	4	4	0
49	1	12	3	17	1	0	1	24	3	0
50	2	0	53	0	0	3	1	3	13	0
51	24	1558	395	2865	20	86	42	392	555	93
52	1	0	4	1	4	8	7	0	0	4

CODE	V.01	V.03	V.04	V.06	V.09	V.10	V.15	V.19	V.24	V.26
53	122	1	80	132	159	1403	223	37	106	290
54	134	9	266	626	4	270	61	10	0	408
55	21	0	0	0	17	0	0	4	0	9
56	0	0	0	0	0	0	0	0	0	0
57	0	0	0	0	0	1	0	16	0	0
58	0	0	0	0	0	7	0	1	4	0
59	0	0	0	0	1	2	2	1289	0	0
60	0	0	0	0	0	1	0	814	0	0
61	40	0	18	20	42	140	47	132	146	29
62	2	1	1	95	32	0	0	173	580	142
63	7	1	0	0	471	138	74	13	247	3
64	236	3213	205	285	928	1400	1347	341	776	359
65	0	0	0	683	205	0	0	73	202	410
66	98	1	23	197	176	614	15	167	390	169
67	117	2056	116	94	426	229	70	281	598	210
68	0	0	15	0	33	0	0	6	35	0
69	6938	513	449	2546	9495	1697	1184	2379	3932	2957
70	0	0	0	2	0	0	0	0	0	5
71	6	0	64	4	43	75	48	13	33	65
72	0	0	0	10	21	22	0	7	6	15
73	0	0	0	0	0	0	0	0	1	1
74	1	0	0	0	145	1	0	188	2	190
75	211	68	384	440	1104	0	163	129	733	772
76	107	119	53	304	459	244	82	186	805	527
77	0	0	0	0	0	10	36	0	0	0

CODE	V.01	V.03	V.04	V.06	V.09	V.10	V.15	V.19	V.24	V.26
78	0	0	0	0	0	13	3	0	0	12
79	16	0	0	7	49	22	9	107	38	8
80	121	236	150	127	8	474	327	3	4	567
81	0	0	0	4	0	0	3	269	0	1
82	1	0	0	0	0	1	0	0	0	0
83	353	2	238	154	445	1583	50	10	75	423
84	674	4	412	946	2580	705	649	818	1143	2527
85	1	0	9	1	0	2	59	1	2	0
86	5923	6	2006	5029	6865	5163	3495	2112	3377	3452
87	0	0	2	3	0	3	0	0	0	0
88	101	0	78	159	456	1147	686	101	388	385
89	1	0	1	0	0	11	4	0	0	0
90	0	0	1	0	0	0	0	0	0	0
91	428	1	109	417	174	71	995	161	85	931
92	161	199	221	551	252	385	0	151	615	516
93	146	0	158	109	30	84	2	2	5	24
94	125	0	0	0	0	0	0	1	12	0
95	40	0	20	8	100	9	0	94	123	23
96	0	0	0	0	0	0	0	2	0	12
97	3646	22	9469	1316	2	486	1256	507	3636	1137
98	11	0	0	0	0	0	0	0	0	0
99	0	1	0	40	2	36	1	656	135	2
100	0	0	0	0	133	0	0	8	0	87
101	109	1	0	35	2242	635	755	499	0	95
102	21	12799	84	572	11	9	47	41	23	104
103	0	0	0	0	5	29	8	0	0	2
104	0	1	2	1	0	1	5	0	5	0

CODE	V.01	V.03	V.04	V.06	V.09	V.10	V.15	V.19	V.24	V.26
105	0	0	12	159	0	0	0	0	7	0
106	0	1	143	126	335	0	0	293	90	404
107	1	0	50	42	30	349	1	88	28	26
108	0	0	1	5	1	12	28	0	6	2
109	61	103	134	121	381	147	59	177	598	189
110	0	1	1	78	60	1	0	10	268	4
111	14	1	2	20	0	6	11	0	0	18
112	0	0	0	0	0	0	0	0	0	0
113	0	0	0	1	0	0	4	5166	4	4
114	0	0	0	0	0	0	0	0	0	1
115	0	0	1	0	0	7	0	10	0	0
116	0	0	0	3	0	3	0	2	0	10
117	0	0	0	2	0	0	1	0	0	0
118	0	0	0	0	0	0	0	1	0	0
119	0	12	0	3	0	0	0	0	0	1
120	605	0	0	0	0	351	0	0	0	0
121	1207	0	76	276	44	856	607	1149	383	33
122	23	1	0	91	0	0	1	243	309	117
123	49	0	0	4	0	28	16	2	0	6
124	0	0	0	18	0	2	0	0	0	0
125	164	1	72	196	10	820	26	85	594	113
126	105	0	172	674	321	218	445	305	1836	552
127	0	0	0	10	0	5	1	0	18	2
128	0	0	0	0	0	0	0	0	0	0
129	0	0	0	0	0	0	0	0	0	0
130	0	0	0	0	0	1	0	0	0	0
131	2	2	3680	1054	0	0	0	307	0	1

CODE	V.01	V.03	V.04	V.06	V.09	V.10	V.15	V.19	V.24	V.26
132	88	1055	1629	620	0	81	12	122	234	13
133	345	2109	140	131	12	390	797	755	1104	53
134	0	8	0	0	0	0	0	0	0	0
135	0	0	0	0	0	0	0	0	0	0
136	3	24	6	5	0	3	2	3	3	0
137	375	7695	60	2825	16	25	8	15	55	634
138	1	6	2	125	1	1	0	2	2	2
139	0	6	1	0	0	1	0	0	2	0
140	5	0	0	0	0	0	0	0	0	0
141	3	0	1	2	0	0	0	0	1	0
142	1	0	1	0	0	0	0	0	0	0
143	0	0	0	0	0	0	0	0	0	0
144	0	0	1	1	0	0	0	0	0	0
145	0	0	0	0	0	3	0	0	0	0
146	0	0	0	0	0	1	0	0	0	0
147	0	0	0	0	0	20	0	0	0	0
148	0	0	0	0	0	0	0	0	0	0
149	0	0	4	0	0	0	0	0	0	0
150	106	0	0	270	429	591	0	0	71	210
151	0	0	0	0	0	0	0	0	0	0
152	0	2	0	1	2	3994	3837	0	2	0
**LABEL**	HIGH	HIGH	HIGH	HIGH	HIGH	HIGH	HIGH	HIGH	HIGH	HIGH

Supplementary Table S4. Bacterial Counts in HIV-infected patients with having 5-10% of activated CD4(+) T cells (MEDIUM)

CODE	V.02	V.05	V.07	V.11	V.12	V.13	V.14	V.16	V.17	V.18	V.20
1	52	0	0	96	14	16	2	156	0	34	0
2	4	0	0	0	0	0	0	0	0	0	0
3	3	11	0	0	12	1	10	20	0	23	12
4	0	0	0	0	0	0	0	0	2	0	0
5	0	0	0	0	0	0	0	1	0	0	0
6	264	64	0	84	336	2	39	7035	122	3031	2
7	0	0	0	0	0	0	0	0	0	0	0
8	0	0	0	0	0	0	0	0	0	0	0
9	4	3	3	4	4	6	0	4	5	4	1
10	0	0	1	0	0	0	0	1	0	0	0
11	0	2	4	1	15	1	0	1	0	6	1
12	0	0	0	0	0	0	0	0	0	0	0
13	0	0	2	0	2	0	4	1	0	2	1
14	31	8	1	682	156	61	141	660	104	198	16
15	0	0	0	1	0	0	0	25	0	0	0
16	25	0	16	4	20	34	36	115	28	21	9
17	34	811	28	133	42	72	54	38	125	127	3
18	0	0	0	4	0	0	0	0	0	0	0
19	0	0	0	0	0	8	0	0	0	0	0
20	5	38	0	13	6	0	24	121	4	12	3
21	186	66	120	1341	92	11862	128	2762	7906	180	101
22	50	0	0	43	0	35	0	35	0	2	0
23	5	0	0	28	8	12	3	17	25	0	0
24	1	11	0	9	0	67	0	7	10	1	3
25	172	32	49	923	24	289	5	485	38	5	10

CODE	V.02	V.05	V.07	V.11	V.12	V.13	V.14	V.16	V.17	V.18	V.20
26	0	0	0	0	0	0	0	0	0	0	0
27	159	0	124	73	325	1	520	0	193	2	1
28	16	0	0	0	9	1179	0	30	23	0	2
29	10912	22865	27491	12055	8284	2086	11683	60	4787	11926	24406
30	0	0	0	0	0	0	0	0	0	0	0
31	55	6	0	987	9	340	14	442	69	4	4
32	262	45	0	0	1055	46	70	0	630	11	67
33	254	987	697	746	624	253	217	58	339	34	5
34	330	0	0	0	83	98	19	0	65	333	0
35	3	0	0	0	121	0	6	0	492	0	8
36	1	14	17	0	0	5	1	136	0	0	0
37	165	1	0	0	0	0	147	0	0	0	0
38	2	1	0	0	0	0	0	2	2	1	0
39	0	0	0	0	0	0	0	1	0	0	0
40	0	2	2	0	4	5	1	8	0	0	13
41	3	9	40	22	9	7	2	12	7	9	6
42	0	0	0	0	0	0	1	0	0	0	0
43	2	0	2	1	3	1	0	3	5	3	2
44	3	1	8	30	0	1	6	1	3	229	0
45	5	2	106	32	362	2	6	2405	310	1121	192
46	0	0	0	0	0	0	0	0	0	0	0
47	0	1	0	0	0	0	0	19	1	3	0
48	0	4	20	315	0	2	0	10	7	18	3
49	0	0	2	0	0	0	3	3	2	1	1
50	0	6	0	4	2	1	1	8	33	5	0
51	5	74	44	89	202	52	48	232	119	1082	436
52	5	3	0	9	2	2	0	2	0	1	0

CODE	V.02	V.05	V.07	V.11	V.12	V.13	V.14	V.16	V.17	V.18	V.20
53	936	209	0	124	280	70	63	184	130	29	3
54	138	2	0	80	39	780	52	155	68	361	103
55	0	0	0	0	8	0	8	0	2	4	0
56	0	0	0	1	0	4	0	0	0	0	0
57	0	0	0	0	0	0	0	0	0	0	0
58	0	0	0	0	0	0	0	0	0	0	0
59	0	0	0	0	0	0	1	2	0	0	0
60	0	0	0	0	0	0	0	1	0	0	0
61	43	13	0	242	16	9	5	363	37	6	2
62	1	0	3	38	22	1	12	110	17	18	2
63	22	168	13	39	91	1	5	98	21	14	1
64	258	237	386	928	597	350	164	2834	912	295	49
65	27	0	0	0	14	0	71	0	168	0	0
66	109	95	0	296	333	45	94	226	214	111	46
67	208	145	235	340	481	152	154	1188	240	178	26
68	12	0	0	0	20	0	13	21	31	2	2
69	2445	1626	297	2233	2777	1060	588	1462	3034	383	238
70	0	0	0	0	0	0	0	0	0	0	3
71	14	320	0	6	25	29	28	7	50	20	2
72	36	1	3	14	13	19	2	12	25	3	1
73	0	0	0	0	1	0	0	0	0	0	0
74	23	0	0	0	40	0	0	0	44	0	2
75	611	633	1722	128	888	713	715	922	385	2175	1069
76	149	373	16	195	69	352	171	28	219	123	50
77	0	0	0	0	0	0	0	0	0	0	0

CODE	V.02	V.05	V.07	V.11	V.12	V.13	V.14	V.16	V.17	V.18	V.20
78	7	0	0	3	0	0	0	0	0	0	0
79	17	2	0	0	16	0	4	685	43	0	0
80	557	649	0	289	23	2770	307	1271	56	315	1
81	0	0	0	1	0	0	0	1	37	0	0
82	0	0	0	0	0	2	0	0	0	0	0
83	472	59	1	1008	35	112	3	4	23	2	1
84	1553	880	292	542	518	1006	745	1239	831	248	430
85	1	2	1	8	0	6	0	6	15	0	0
86	10741	2620	18	3661	2287	1326	1009	1100	1267	1417	159
87	5	0	0	9	0	0	0	0	0	0	0
88	410	216	17	213	279	198	110	139	183	33	59
89	0	0	0	1	1	4	0	1	0	0	0
90	0	0	0	0	0	0	0	0	0	0	0
91	756	390	0	29	164	273	430	100	2543	79	13
92	516	187	1401	223	222	282	427	937	505	98	53
93	62	26	0	7	48	0	73	0	0	2	0
94	0	4	0	2	0	0	0	165	0	398	0
95	23	9	17	36	99	0	31	11	32	21	12
96	0	0	0	0	0	0	0	0	0	0	0
97	2896	2070	0	3672	3740	4093	412	1337	652	4150	274
98	0	0	0	0	0	0	0	0	0	0	0
99	0	0	0	157	213	0	57	0	3	1876	17
100	0	0	0	422	102	0	25	0	20	10	0
101	89	2	0	9	541	0	59	129	100	1	23
102	14	9	10	1	60	14	11	3	1	38	77
103	6	0	0	1	0	2	0	0	0	0	0
104	0	2	1	0	1	1	0	0	0	0	0

CODE	V.02	V.05	V.07	V.11	V.12	V.13	V.14	V.16	V.17	V.18	V.20
105	0	0	0	0	0	0	0	0	0	0	0
106	753	293	30	0	883	81	588	2867	1023	1236	68
107	116	43	5	451	11	2	48	1	62	24	2
108	0	1	0	8	0	0	0	1	0	0	1
109	668	115	71	1770	99	3	183	197	369	230	18
110	0	1	1	0	82	0	14	205	294	0	0
111	64	1	1	2	4	139	6	147	3	0	0
112	0	0	0	0	0	0	0	0	0	0	0
113	0	0	0	0	0	0	1	1	8	1	0
114	0	0	0	0	0	0	0	0	0	0	0
115	0	1	0	0	0	0	2	0	0	0	0
116	13	0	0	0	0	0	0	0	0	0	0
117	0	1	0	0	0	0	0	0	0	0	0
118	0	0	0	0	128	0	0	0	0	0	0
119	0	0	0	0	0	0	1	1	0	0	1
120	0	0	0	4	0	0	2	0	1	2	0
121	33	343	0	80	44	51	446	15	13	6	120
122	0	38	0	0	0	0	15	0	0	10	0
123	33	11	0	35	82	25	4	0	13	0	0
124	0	0	0	0	14	0	1	0	0	0	22
125	17	55	0	116	1	0	0	76	12	2	3
126	371	513	0	205	80	88	156	40	126	15	0
127	0	1	0	0	0	0	0	1	0	1	6
128	0	0	0	0	0	0	0	0	0	0	32
129	0	0	0	0	0	0	0	0	0	0	0
130	0	0	0	1	0	0	0	0	0	0	0
131	3303	1	0	0	1376	3	11444	1	2274	1076	26

CODE	V.02	V.05	V.07	V.11	V.12	V.13	V.14	V.16	V.17	V.18	V.20
132	34	11	64	11	12	167	1	39	150	226	7
133	51	25	537	251	37	1249	29	92	1725	116	33
134	0	0	0	0	0	0	0	0	0	0	0
135	0	0	0	3	0	0	0	0	0	0	0
136	1	0	2	0	0	9	0	1	26	2	0
137	24	319	7	1	1064	369	180	2	122	415	1463
138	0	1	0	0	0	42	7	1	0	0	29
139	0	0	0	0	1	0	0	0	0	0	0
140	0	0	0	0	0	0	0	0	0	0	0
141	0	0	0	0	1	2	1	1	1	0	0
142	0	1	0	0	0	0	1	0	0	0	0
143	1	0	0	1	0	0	1	0	2	1	0
144	1	0	0	0	0	0	0	0	1	0	0
145	4	0	0	0	0	22	1	0	0	0	0
146	0	0	0	1	2274	0	0	0	0	0	0
147	3	0	0	0	0	56	0	0	0	1	0
148	0	0	0	0	1	0	0	0	0	0	0
149	0	0	0	0	0	0	0	0	0	0	0
150	586	194	0	1060	35	0	17	0	65	1	1
151	5	0	0	0	0	0	0	0	0	0	0
152	24	2	0	0	0	35	1	4	0	19	0
**LABEL**	MEDIUM	MEDIUM	MEDIUM	MEDIUM	MEDIUM	MEDIUM	MEDIUM	MEDIUM	MEDIUM	MEDIUM	MEDIUM

Supplementary Table S5. Bacterial Counts in HIV-infected patients with having less than 5 of activated CD4(+) T cells (LOW)

CODE	V.08	V.23	V.25	V.27
1	0	136	0	0
2	0	0	0	0
3	12	51	9	4
4	0	0	0	0
5	0	0	0	0
6	106	1	31	56
7	0	0	0	0
8	0	1	2	0
9	1	6	3	3
10	0	0	0	0
11	1	1	1	0
12	0	3	0	0
13	7	8	1	1
14	13	38	55	109
15	0	1	11	0
16	2	1	0	21
17	3	38	4	63
18	0	1	0	0
19	0	0	0	0
20	1	1	0	5
21	189	5233	21939	97
22	20	3	0	0
23	13	27	19	0
24	3	80	17	2
25	45	412	2535	44

CODE	V.08	V.23	V.25	V.27
26	0	0	0	0
27	0	1438	0	43
28	2	0	0	0
29	24256	13480	2	30736
30	0	0	0	0
31	9	39	1126	7
32	28	2415	0	0
33	7	2	3	5
34	1	0	0	1
35	17	0	0	0
36	1	9	1	0
37	1	0	0	0
38	1	0	0	0
39	0	0	0	0
40	1	2	1	2
41	4	2	6	9
42	0	0	0	0
43	1	1	3	3
44	0	11	3	2
45	3	41	1	68
46	0	0	0	0
47	0	1	0	0
48	0	1	0	0
49	0	9	1	0
50	1	1	0	0
51	16	49	142	174
52	1	0	0	0

CODE	V.08	V.23	V.25	V.27
53	9	60	0	1
54	26	70	48	2660
55	0	0	0	0
56	0	0	0	0
57	0	0	0	0
58	0	0	0	0
59	0	0	0	0
60	0	0	0	0
61	3	15	4	0
62	0	4	0	10
63	17	23	32	6
64	259	97	171	114
65	90	0	0	0
66	71	19	15	45
67	76	94	29	106
68	0	1	0	0
69	1155	781	328	129
70	3	0	0	0
71	53	103	103	0
72	0	1	0	0
73	0	0	0	0
74	0	0	0	0
75	490	928	536	312
76	452	120	336	309
77	0	0	0	0

CODE	V.08	V.23	V.25	V.27
78	0	0	0	0
79	0	36	0	2
80	8	44	6	562
81	1	2	0	0
82	0	0	0	0
83	28	5	1	2
84	472	396	2	309
85	0	0	22	0
86	452	1239	92	29
87	0	0	0	0
88	199	57	120	17
89	2	1	0	0
90	0	0	0	0
91	458	97	79	5
92	64	110	172	67
93	20	3	0	0
94	0	6	722	0
95	7	4	5	23
96	0	0	0	0
97	594	1054	1	653
98	0	0	0	0
99	0	0	0	0
100	0	0	0	0
101	0	0	77	91
102	6	8	2	15
103	0	0	0	0
104	0	1	2	3

CODE	V.08	V.23	V.25	V.27
105	0	0	0	0
106	61	0	0	214
107	11	14	0	2
108	0	0	0	1
109	34	0	4	51
110	0	7	0	0
111	1	0	0	0
112	0	0	0	0
113	0	3	62	0
114	0	0	0	0
115	0	0	0	0
116	0	0	1	0
117	0	0	0	0
118	0	0	0	0
119	0	1	0	0
120	0	1513	0	0
121	288	30	942	476
122	2	0	0	5
123	0	0	0	0
124	0	0	0	0
125	4	77	144	0
126	12	0	0	7
127	676	0	0	6
128	0	0	0	0
129	0	0	0	0
130	0	0	0	0
131	0	1	1	610

CODE	V.08	V.23	V.25	V.27
132	50	14	0	6
133	7	340	2	15
134	0	0	0	0
135	0	0	0	0
136	0	1	1	0
137	0	16	0	90
138	0	0	0	1
139	0	2	0	0
140	0	0	0	0
141	1	0	0	0
142	0	0	0	0
143	1	0	0	0
144	0	0	1	0
145	0	0	0	0
146	0	0	0	0
147	0	0	0	0
148	4	0	0	0
149	0	0	0	0
150	2	128	0	1
151	0	0	0	0
152	0	0	3657	4
**LABEL**	LOW	LOW	LOW	LOW

Supplementary Table S6. Bacterial Counts in control group (< 5% of activated CD4(+) T cells)

CODE	C.01	C.02	C.04	C.05	C.09	C.10	C.14	C.23	C.24	C.25	C.26	C.27
1	48	0	0	735	1	19	20	0	9	0	0	1
2	0	0	0	0	0	0	0	0	0	0	0	0
3	4	35	4	2	9	35	10	64	49	26	7	33
4	0	0	0	13	0	0	0	0	0	0	0	0
5	0	0	0	0	0	0	0	0	0	0	0	0
6	836	132	5	17	17	115	171	13772	69	847	113	1529
7	0	0	0	0	0	0	0	0	0	0	0	0
8	1	0	0	0	0	1	0	0	1	1	0	4
9	1	6	4	3	3	2	6	12	0	10	8	3
10	0	0	1	0	0	0	0	0	0	0	0	1
11	0	0	0	1	2	4	1	10	7	0	0	48
12	0	0	0	1	0	14	0	0	0	0	36	0
13	1	6	3	0	1	2	1	6	20	6	0	7
14	33	67	3	23	18	32	13	110	17	0	0	322
15	0	0	0	0	0	0	0	13	0	8	3	0
16	21	17	0	16	1	125	8	0	9	0	3	56
17	58	49	2	20	18	120	8	24	7	91	21	442
18	0	0	0	0	0	0	0	2	0	0	3	0
19	0	0	0	0	0	0	0	0	0	3	0	0
20	2	10	2	3	5	17	4	1	0	0	0	16
21	2252	1165	179	1746	80	7006	54	766	3552	22110	8418	239
22	61	4	0	0	2	1584	0	1	22	77	1041	1
23	12	33	0	5	0	50	3	2	13	86	97	44
24	47	9	0	36	1	57	1	0	7	0	309	1
25	354	94	0	18	18	2860	14	49	1295	1517	1426	69

CODE	C.01	C.02	C.04	C.05	C.09	C.10	C.14	C.23	C.24	C.25	C.26	C.27
26	0	1	0	1	0	0	0	0	0	0	1	0
27	30	1836	261	649	973	0	614	0	793	0	0	306
28	1	60	0	0	0	1211	12	0	52	0	492	0
29	1944	15896	20795	4757	20068	8620	17566	6	11157	1	640	2716
30	0	0	0	0	0	0	10	0	0	0	0	0
31	37	105	0	13	1	428	4	21	207	3489	3265	30
32	1065	336	12	934	3	685	77	0	0	0	0	887
33	140	335	4	4	56	5	115	7	201	11	29	101
34	39	841	20	0	61	0	31	0	0	0	0	0
35	447	397	0	213	0	0	4	0	0	0	6	0
36	0	1	1	2	0	22	1	13	0	1	3	3
37	0	189	0	133	0	0	83	0	0	0	0	0
38	1	1	0	0	1	0	1	3	0	0	2	1
39	0	0	0	0	1	0	0	0	0	0	0	0
40	1	6	0	2	0	3	3	3	18	0	2	2
41	2	6	5	5	4	1	10	8	4	9	5	12
42	0	0	0	0	0	0	0	0	3	0	0	0
43	0	2	1	0	0	1	3	0	1	2	2	1
44	1	0	54	0	0	114	12	57	8	5	29	21
45	264	208	20	5	15	30	31	21	16	152	3	251
46	0	0	0	0	0	0	0	10	0	0	0	0
47	0	0	0	0	0	1	0	0	1	0	0	0
48	2	0	0	0	0	35	0	202	43	0	6	3
49	0	1	0	1	3	2	8	5	14	0	1	2
50	0	1	0	0	0	18	2	80	1	1	5	12
51	7	56	95	4	259	142	638	2219	5185	297	8	30
52	2	4	0	2	0	0	1	0	1	0	2	3

CODE	C.01	C.02	C.04	C.05	C.09	C.10	C.14	C.23	C.24	C.25	C.26	C.27
53	164	124	0	155	6	200	70	9	15	0	944	460
54	28	595	75	257	713	841	388	5	13	37	222	89
55	1	10	1	0	0	0	1	0	0	0	0	0
56	0	0	0	0	0	0	0	0	0	1	0	0
57	0	0	0	0	0	0	0	0	0	0	0	0
58	0	0	0	2	0	0	0	0	0	0	0	0
59	0	0	0	1	0	0	0	0	0	0	0	1
60	0	0	0	3	0	0	0	0	0	0	0	0
61	14	11	0	39	0	34	2	2	17	3	41	68
62	6	3	0	18	2	15	7	9	10	7	0	285
63	5	0	0	97	0	59	58	621	3	32	60	33
64	171	170	21	58	21	383	92	943	57	151	318	1339
65	0	568	0	260	0	0	29	1	2	0	3	264
66	89	195	3	56	60	434	28	10	38	57	93	1023
67	94	79	7	50	34	186	27	1048	72	186	46	876
68	1	10	0	10	0	14	2	0	1	0	0	20
69	422	851	35	555	288	1307	607	2670	268	276	1749	1402
70	0	0	0	0	0	0	0	0	0	0	0	0
71	10	30	76	19	22	72	54	0	137	9	24	14
72	5	1	0	2	0	16	0	4	0	0	5	14
73	0	0	0	0	0	0	0	0	0	0	0	0
74	14	53	1	0	1	169	31	1	3	0	0	0
75	866	322	107	667	158	641	68	4	369	200	465	4374
76	22	92	37	276	109	492	13	1	468	18	313	123
77	0	0	0	0	0	0	0	0	0	0	0	0

CODE	C.01	C.02	C.04	C.05	C.09	C.10	C.14	C.23	C.24	C.25	C.26	C.27
78	4	0	0	3	0	0	0	0	0	0	0	0
79	47	41	0	55	1	90	0	0	7	0	7	581
80	3	196	17	260	368	757	117	316	16	62	289	18
81	1	0	0	1	0	0	0	0	0	0	0	4
82	0	0	0	0	0	0	0	0	0	0	2	0
83	635	204	0	381	0	145	4	0	17	22	100	128
84	95	753	54	448	200	743	349	716	333	210	626	4992
85	3	1	0	0	0	7	2	3	0	84	2	0
86	3678	2504	17	2597	209	4467	557	181	1467	925	3653	4213
87	0	0	0	0	0	0	0	0	0	0	0	0
88	45	70	0	118	26	469	24	1454	41	149	180	535
89	0	2	0	2	0	0	0	0	0	0	3	0
90	0	0	0	0	0	0	0	0	0	0	1	0
91	172	697	0	325	11	85	152	3	12	235	964	441
92	188	117	3	65	47	389	243	3504	62	334	528	1343
93	11	58	0	139	0	30	4	0	0	0	28	12
94	0	0	7	0	35	0	15	88	529	1722	0	61
95	2	13	1	11	13	15	5	4	86	18	5	19
96	0	0	977	0	188	0	3	0	0	0	0	0
97	4	1164	196	1	1234	671	140	233	1376	342	0	1968
98	0	0	41	0	0	0	0	0	0	0	0	0
99	1502	1	282	0	1018	0	148	2	63	303	0	177
100	15	142	11	0	70	0	3	0	0	0	0	39
101	174	7	27	224	10	1	36	4	0	88	1779	137
102	6	26	143	9	142	66	628	107	221	15	12	20
103	0	0	0	0	0	0	0	0	0	0	10	0
104	0	2	2	1	1	1	2	4	0	4	1	2

CODE	C.01	C.02	C.04	C.05	C.09	C.10	C.14	C.23	C.24	C.25	C.26	C.27
105	0	237	0	0	0	0	0	1	0	0	0	0
106	576	177	346	86	30	0	79	181	183	0	0	756
107	0	35	0	13	0	24	14	1414	0	4	114	46
108	1	1	0	0	0	1	0	0	1	0	3	0
109	127	123	14	88	12	116	22	25	18	2	8	489
110	34	16	1	7	26	72	5	3	12	2	0	351
111	2	1	6	29	5	1	36	2	0	1	20	1
112	0	0	0	0	0	0	10	0	0	0	0	17
113	0	0	12	1	0	0	4	2	225	0	0	2
114	0	0	0	0	0	0	5	0	0	0	0	0
115	0	0	0	0	0	0	0	0	0	0	1	0
116	0	5	0	14	0	0	0	0	0	0	0	0
117	0	0	0	0	0	0	0	0	0	0	0	0
118	16	110	0	0	0	0	0	0	0	0	21	0
119	0	0	0	0	0	0	0	0	0	0	0	0
120	0	0	0	1	0	11	0	0	15	0	0	0
121	14	30	284	238	21	195	31	124	197	1819	75	25
122	0	0	0	313	32	3	7	0	0	0	0	0
123	14	6	0	19	0	0	0	0	0	0	12	1
124	0	0	0	0	0	0	2	0	0	0	0	0
125	18	12	0	8	4	19	0	0	16	319	301	11
126	123	597	2	28	10	14	46	2	597	9	982	538
127	2	0	0	0	1	0	3	0	2	0	1	0
128	0	0	0	0	0	0	0	0	0	0	0	0
129	8	0	0	0	0	0	0	0	0	0	0	0
130	0	0	0	4318	0	0	0	0	0	0	0	0
131	12532	6691	4967	10271	1423	2	353	2	0	3	0	273

CODE	C.01	C.02	C.04	C.05	C.09	C.10	C.14	C.23	C.24	C.25	C.26	C.27
132	4	31	47	2	39	374	688	553	700	6	186	2
133	21	134	19	633	32	619	2163	5478	122	40	1247	35
134	0	0	0	0	0	0	79	0	0	0	0	0
135	0	0	0	0	0	0	0	0	0	0	0	0
136	0	1	0	0	0	11	43	23	4	0	4	0
137	4	63	1936	20	1862	121	4012	129	534	1	452	46
138	1	0	25	0	22	2	151	0	22	0	1	0
139	0	2	2	0	0	3	0	1	0	0	0	1
140	0	0	0	0	0	0	0	0	0	0	0	0
141	0	3	0	0	0	1	1	2	0	1	1	0
142	1	0	0	0	0	0	0	0	1	0	1	0
143	0	0	0	0	0	0	2	0	1	0	1	0
144	0	0	0	0	0	0	0	0	0	0	1	1
145	9	0	0	188	0	0	0	0	0	0	1	0
146	0	0	0	0	0	0	0	0	0	0	0	1
147	0	0	0	0	0	0	0	3	0	0	0	0
148	0	0	0	1	0	0	0	0	0	0	0	0
149	0	0	0	0	0	0	0	0	0	0	0	0
150	858	75	0	61	0	4	10	0	0	0	105	439
151	0	0	0	0	0	0	0	0	0	0	0	0
152	1	0	0	94	1	71	0	131	0	1	1747	1
**LABEL**	Control	Control	Control	Control	Control	Control	Control	Control	Control	Control	Control	Control

Supplementary Table S7. Tukey least significance difference test comparing the % of activated CD4(+) T cells by HIV-infected group.

Groups	Difference	95%CI	*P*
Medium vs. High	-9.465	(-13.134, -5.7949)	**<0.00001**
Low vs. High	-13.635	(-18.603, -8.6663)	**<0.00001**
Control vs. High	-13.385	(-16.981, -9.7889)	**<0.00001**
Low vs. Medium	-4.170	(-9.0741, 0.73321)	0.119
Control vs. Medium	-3.920	(-7.4261, -0.4147)	**0.024**
Control vs. Low	0.250	(-4.5988, 5.09887)	0.999

Conventions as in Figure 3A of the main manuscript.

Supplementary Table S8. Hits with having the adjusted P values < 0.05 after MetagenomeSeq analysis between Control group and HIV-infected group

Family	Genus	F	P	Adjusted P
Lachnospiraceae	Incertae_Sedis	21.2078968	1.0877E-06	0.0001675
Lachnospiraceae	Blautia	18.9666402	3.0257E-06	0.00022861
Veillonellaceae	Veillonella	18.1545952	4.4535E-06	0.00022861
Lachnospiraceae	Dorea	12.7155631	7.6708E-05	0.00295326
Enterobacteriaceae	Escherichia-Shigella	11.1999067	0.00018623	0.00573583
Ruminococcaceae	Faecalibacterium	10.7837297	0.00023959	0.00614938
Corynebacteriaceae	Corynebacterium	8.59304841	0.00096387	0.01508861
Staphylococcaceae	Staphylococcus	8.77304486	0.00085589	0.01508861
Ruminococcaceae	g	8.56834664	0.00097978	0.01508861
Ruminococcaceae	Subdoligranulum	8.37904194	0.00111133	0.01555867
Streptococcaceae	Streptococcus	7.91041585	0.00152433	0.01956227
Pasteurellaceae	Actinobacillus	7.78096423	0.00166513	0.01972533
Rickettsiales_mitochondria	g	7.33338919	0.00226815	0.02494962
Enterobacteriaceae	Enterobacter	6.79073394	0.00332491	0.03413574
Erysipelotrichaceae	Incertae_Sedis	6.23360347	0.00496973	0.04783362
Lachnospiraceae	Shuttleworthia	6.09954775	0.00548228	0.04966303

Highlighted in yellow are those genera identified in the sPLS-DA signature

Supplementary Table S9. Top-half of the hits with having the adjusted P values < 0.05 after MetagenomeSeq analysis between Control group and HIV-infected subgroup with HIGH % activated CD4(+) T cells

Family	Genus	F	P	Adjusted P
Lachnospiraceae	Incertae_Sedis	92,8999628	1,9168E-14	2,9518E-12
Lachnospiraceae	Blautia	59,7686836	8,1982E-12	6,3126E-10
Lachnospiraceae	Dorea	41,404943	8,315E-10	4,2684E-08
Ruminococcaceae	Faecalibacterium	36,4985052	3,6644E-09	1,4108E-07
Lachnospiraceae	Pseudobutyrivibrio	35,0729114	5,7849E-09	1,7817E-07
Veillonellaceae	Veillonella	31,5716447	1,8767E-08	4,8169E-07
Ruminococcaceae	Subdoligranulum	29,4792315	3,9517E-08	7,6071E-07
Ruminococcaceae	g	28,1246568	6,5167E-08	1,1151E-06
Staphylococcaceae	Staphylococcus	26,0620322	1,4378E-07	2,2143E-06
Lachnospiraceae	Roseburia	25,1993636	2,0248E-07	2,8347E-06
Corynebacteriaceae	Corynebacterium	23,8089322	3,569E-07	4,5802E-06
Streptococcaceae	Streptococcus	23,074759	4,8522E-07	5,748E-06
Enterobacteriaceae	Escherichia-Shigella	21,9533261	7,8434E-07	8,6278E-06
Alcaligenaceae	Sutterella	20,0713289	1,8132E-06	1,8616E-05
Bacteroidaceae	Bacteroides	19,4970641	2,3616E-06	2,273E-05
Ruminococcaceae	Incertae_Sedis	18,3779986	4,0007E-06	3,6241E-05
Peptostreptococcaceae	Incertae_Sedis	15,9014866	1,3669E-05	0,00011694
Actinomycetaceae	Actinomyces	15,7345564	1,4898E-05	0,00012075
Pasteurellaceae	Actinobacillus	15,5249466	1,661E-05	0,0001279
Enterobacteriaceae	Enterobacter	13,7640698	4,2633E-05	0,00031264
Erysipelotrichaceae	Incertae_Sedis	13,6022244	4,6617E-05	0,00032632
Clostridiaceae	Clostridium	13,0542316	6,331E-05	0,0004239
Lachnospiraceae	Lachnospira	11,7263751	0,00013612	0,00087346
Prevotellaceae	Prevotella	11,3213833	0,00017315	0,00106192

Highlighted in yellow are those genera identified in the sPLS-DA signature
